# Supplementary material for: Platanus-allee is a de novo haplotype assembler enabling a comprehensive access to divergent heterozygous regions
Source: Nat Commun. 2019 Apr 12;10:1702. doi: 10.1038/s41467-019-09575-2 (PMC6461651; doi:10.1038/s41467-019-09575-2)
Supplement: Supplementary file 1 — Supplementary Information [file 41467_2019_9575_MOESM1_ESM.pdf]

Supplementary Information

Platanus-allee is a *de novo* haplotype assembler enabling a comprehensive access to divergent heterozygous regions

Authors

Rei Kajitani[1], Dai Yoshimura[1], Miki Okuno[1], Yohei Minakuchi[2], Hiroshi Kagoshima[3], Asao Fujiyama[3], Kaoru Kubokawa[4], Yuji Kohara[3], Atsushi Toyoda[2,3], Takehiko Itoh[1]

[1] School of Life Science and Technology, Tokyo Institute of Technology, Meguro-ku, Tokyo, 152-8550, Japan

[2] Comparative Genomics Laboratory, National Institute of Genetics, Mishima, Shizuoka, 411-8540, Japan

[3] Advanced Genomics Center, National Institute of Genetics, Mishima, Shizuoka, 411-8540, Japan

[4] Research Center for Marine Education, Ocean Alliance, The University of Tokyo, Bunkyo-ku, Tokyo 113-0033, Japan

Correspondence should be addressed to T.I. (takehiko@bio.titech.ac.jp).

Supplementary Notes 6
Supplementary Note 1. Scaffolding to extend each haplotype sequence.............. 6
Supplementary Note 2. Divisions of scaffolds in error-candidate positions using mapped read information. 7
Supplementary Note 3. Removal of low-confidence edges in scaffold graph. 9
Supplementary Note 4. Divisions of non-bubble nodes in scaffold graph or gapped de Bruijn graph. 9
Supplementary Note 5. Trimming of sparse edge-regions of nodes in scaffold graph. 10
Supplementary Note 6. Iteration of haplotype phasing....................................... 10
Supplementary Note 7. Description of the algorithms of FALCON-Unzip and Supernova. 16
Supplementary Note 8. Pre-processes of reads. 16
Supplementary Note 9. Estimation of the genome size from short-reads. 17
Supplementary Note 10. Execution of Platanus-allee. 17
Supplementary Note 11. Execution of Supernova. 17
Supplementary Note 12. Execution of FALCON-Unzip. 18
Supplementary Note 13. Division of the primary contigs of FALCON-Unzip to make the phased block set. 23
Supplementary Note 14. Execution of Pilon. 24
Supplementary Note 15. Execution of Purge-Haplotigs. 24
Supplementary Note 16. Execution of GenomeScope. 25
Supplementary Note 17. Counting exactly matched mate-pairs for phased blocks in the P. polytes benchmark. 25
Supplementary Note 18. Calculation of the numbers of switch-errors and mis-assemblies for the C. elegans data. 25
Supplementary Note 19. Calculation of the numbers of mis-assemblies in consensus sequences for the C. elegans data. 26
Supplementary Note 20. Depiction of dot plots of sequence alignments. 27
Supplementary Note 21. Depiction of alignments as lines. 27
Supplementary Note 22. Construction of the phased blocks of the pipeline of Mostovoy et al. (2016)[15] 27

Supplementary Note 23. Evaluation of phased blocks of the human NA12878 using the Platinum variant set. 28
Supplementary Note 24. Evaluation of the MHC region of the human NA12878 using the previous typing result. 29
Supplementary Note 25. Search of HLA-related genes for the human NA12878 phased blocks. 31
Supplementary Note 26. Benchmarking for the other organisms using the public data. 31
Supplementary Note 27. Benchmarking for fully simulated heterozygous data. 32
Supplementary Note 28. Calculation of precision and recall for the Arabidopsis thaliana F1-hybrid benchmark. 34
Supplementary Note 29. Measurement of run time of Platanus-allee. 34
Supplementary Note 30. Descriptions of the costs of sequencing technologies... 34
Supplementary Note 31. Previous public data used in this study. 35

Supplementary Tables 39
Supplementary Table 1. Sequencing reads of P. polytes (swallowtail butterfly). 39
Supplementary Table 2. Sequencing reads of B. japonicum (amphioxus). 40
Supplementary Table 3. Sequencing reads of C. elegans (N2 and CB4856 strains). 41
Supplementary Table 4. Sequencing reads of H. sapiens (human) NA12878 . 42
Supplementary Table 5. Heterozygosities estimated by GenomeScope based on k-mer information. 43
Supplementary Table 6. NG50 of primary contigs of FALCON-Unzip for various parameters. 44
Supplementary Table 7. Phased block statistics for various input data. 45
Supplementary Table 8. Consensus sequence statistics for various input data. 46
Supplementary Table 9. Real run time of Platanus-allee. 47
Supplementary Table 10. CPU run time of Platanus-allee. 47
Supplementary Table 11. Evaluation of B. japonicum phased blocks based on synthetic long reads. 48
Supplementary Table 12. Evaluation of phased blocks of the synthetic diploid sample (C. elegans) based on the reference genomes. 49

Supplementary Table 13. Evaluation of the errors in phased blocks for C. elegans synthetic diploid data. 50
Supplementary Table 14. Accuracy evaluation of consensus scaffolds for C. elegans synthetic diploid data. 51
Supplementary Table 15. Evaluation of H. sapiens phased blocks based on synthetic long reads. 52
Supplementary Table 16. Evaluation of human NA12878 phased blocks based on the Platinum data (Eberle et al., 2017[16]). 53
Supplementary Table 17. Evaluation of the errors in phased blocks for human NA12878 based on the Platinum data (Eberle et al., 2017[16]). 54
Supplementary Table 18. Evaluation of MHC (HLA) loci of human NA12878 based on the previous typing results (Dilthey et al., 2016[17]). 55
Supplementary Table 19. Platanus-allee bubbles not found in the reference and the other assemblies for human data (NA12878) 56
Supplementary Table 20. Sequencing reads of A. thaliana F1-hybrid from public databases. 57
Supplementary Table 21. Sequencing reads of P. yedonensis (cherry blossom) from public databases. 58
Supplementary Table 22. Sequencing reads of P. alecto (bat) from public databases. 59
Supplementary Table 23. Phased block statistics for benchmarks using public data. 60
Supplementary Table 24. Consensus sequence statistics for benchmarks using public data. 62
Supplementary Table 25. Evaluation of A. thaliana (F1 hybrid) phased blocks. 64
Supplementary Table 26. Phased block statistics for fully simulated data of C. elegans. 65
Supplementary Table 27. Evaluation of phased blocks for fully simulated data of C. elegans based on the reference genome. 66

Supplementary Figures 67
Supplementary Fig. 1. Distributions of the numbers of 32-mer occurrences. 67
Supplementary Fig. 2. Schematic model of cross structures and untangling. 68

Supplementary Fig. 3. Untangling cross structure using linked-reads.............. 69
Supplementary Fig. 4. Haplotype synteny-based correction. 71
Supplementary Fig. 5. Schematic model of error detection for benchmarks....... 72
Supplementary Fig. 6. Moleculo contig alignment to the longest bubble absent in the reference (human).. 73
Supplementary Fig. 7. Examples of highly divergent and repeat-rich bubbles of Platanus-allee (human). 75

Supplementary References..76

Supplementary Notes

Supplementary Note 1. Scaffolding to extend each haplotype sequence.

The scaffolding module is derived from that of Platanus[1]. Similar to the contig-assembly module, the bubble-removal function is omitted. As an extension, Platanus-allee can accept the data from long-reads and handle multiple libraries simultaneously. For the derivation, let $r_{\text{tolerance}}$ be the factor that determines the tolerance and k be the mer-length of the input de Bruijn graph for phasing. For multiple pair-end (mate-pair) libraries (n = the number of library), let d_i and a_i be the average and standard deviation of the insert-size of i-th library, respectively. For long-reads and multiple libraries, the tolerance of overlaps to form the layout, $l_{\text{tolerance}}$, is defined as follows:

$$l_{\text{tolerance}} = \begin{cases} r_{\text{tolerance}} \times 2 \times k & \text{(for long reads)} \\ r_{\text{tolerance}} \times \min(d_n,\ a_n/10) & \text{(for multiple libraries)} \end{cases} \quad (1)$$

The cutoff of input sequence lengths is $l_{\text{tolerance}}/2$, which is determined in the same manner as in Platanus. Additionally, the scaffolding module is modified to reduce conflict caused by bubble structures in the scaffold graph. Here, nodes containing anchor bubbles are referred to as "bubble nodes" and are paired according to anchor bubble information. The definition of confliction of nodes is the same as that in Platanus; if the positions of two nodes from the common one node (source node) overlap with a length longer than the tolerance, these two nodes are treated as a conflicting pair. The modified procedures are as follows:

(1) Suppose a bubble node, b, is a source that connects conflicting nodes. If one of the conflicting nodes, c, is the counterpart of the source node, the edge between b and c is deleted from the scaffold graph. This procedure is expected to reduce erroneous conflictions caused from bubbles.

(2) Platanus treats source nodes connecting conflicting nodes as those corresponding to repetitive sequences. An exception is for Platanus-allee, where a source node is not

treated as a repetitive node if the conflicting nodes are bubble nodes that pair with each other. The aim of this modification is the same as that in step (1).

(3) Bubble nodes are not excluded even if their lengths are less than the threshold. This modification is designed to efficiently use informative nodes to phase heterozygous regions.

Supplementary Note 2. Divisions of scaffolds in error-candidate positions using mapped read information.

This function is derived from that of Platanus and is designed to divide nodes of gapped de Bruijn or scaffold graph at candidate positions of mis-assemblies. Note that each node of the graph is a sequence that consists of contigs and gaps, and divisions can be used for gaps. This function is executed in the following two manners:

(1) Division of mis-assembly candidates

Briefly, mis-assembly candidates are internal gaps near which mapped reads support alternative links to other nodes. First, the pairs of end-positions of mapped reads linking different nodes are extracted and used in the following steps. Let l_{average} be the average insert-size of the paired-ends (mate-pairs) library or read-length of the long-reads library and $l_{\text{tolerance}}$ be the tolerance of overlaps for scaffolding (**Supplementary Note 1**). For each pair of end-positions of mapped reads and a gap, let d_{gap} be the distance between the mapped end- position and gap, and d_{edge} be the distance between the mapped end-position and node edge for the direction of the potential link, *i.e.*, the same direction of the mapped read for paired-ends or long-reads, and vice versa for mate-pairs. For each gap located in that direction from the mapped end-position, the number of alternative links, $n_{\text{alt-link}}$, is incremented if

$$d_{\text{gap}} \leq l_{\text{average}} \text{ and } d_{\text{edge}} > l_{\text{average}} + l_{\text{tolerance}} \quad (2).$$

Let $n_{\text{min-link}}$ be the minimum number of links for scaffolding (default number of links is, 3) and k be the mer-length of the input de Bruijn graph for phasing. Finally, the node is divided at the gap if the following condition is satisfied:

$$n_{\text{alt-link}} \geq n_{\text{min-link}} \text{ and gap-size} \geq k \quad (3)$$

Note that the expected number of links is calculated and considered in the corresponding function of Platanus. In contrast, Platanus-allee handles diploid haplotypes, which causes many multiple-hits in the mapping of the reads and makes the calculation of the expected number of links difficult. Thus, we applied the relatively simple requirement above for the divisions.

(2) Division of switch-error candidates

A switch-error is an error of haplotype phasing that results in the mis-joining of paternal and maternal haplotypes. This can occur even if the assembled haplotype sequences are correct as the genomic structure and cannot be corrected by haplotype synteny-based correction (see Methods, Haplotype synteny-based correction). This function aims to divide the node at the positions of such errors.

Here, "bubble-node pairs" are pairs of nodes defined according to the anchor bubbles (for details, Methods, Haplotype synteny-based correction). The definitions of l_{average}, $l_{\text{tolerance}}$, and $n_{\text{min-link}}$ are the same as those in step (1). First, each pair of end-positions of mapped reads linking one node of a bubble-node pair and its counterpart is extracted and used in the following steps. Additionally, end-positions that are not in anchor bubbles are discarded. For one of each end-position pair, the position of the counterpart is converted according to the anchor bubble information and the insert-size is estimated. If this insert-size $\leq l_{\text{average}} + l_{\text{tolerance}}$, the number of links supporting a switch-error, $n_{\text{switch-link}}$, is increased for each gap flanked by the end-positions. In a similar manner, the number of spanning end-position pairs that do not support switch-errors, n_{spanning}, is calculated for each gap. If the following condition is satisfied:

$$n_{\text{switch-link}} > n_{\text{spanning}} \text{ and } n_{\text{switch-link}} \geq n_{\text{min-link}} \quad (4)$$

the node is divided at the gap. After the divisions of (1) or (2), information from which the contigs originated are stored and used to prevent the recurrences of errors in the next scaffolding step. Specifically, links between the contigs included in the same node and divided into the separate nodes are deleted from the scaffolding step.

Supplementary Note 3. Removal of low-confidence edges in scaffold graph.

This function is derived from that of Platanus. The function removes erroneous edges that cause conflicts in a scaffold graph. The purpose of this function is to simplify the graph and improve the extension of scaffolds. Supposing that two nodes connected to the common node (source node) are conflicting (Supplementary Note 1), removal of the edges can be executed according to the number of links or to the barcode information of linked reads.

(1) According to the number of links

Let the number of links supporting the edges between the source node and conflicting nodes be n_1 and n_2. If $n_1 > 8 \times n_2$, the edge corresponding to n_2 is removed. The expected number of links is calculated and used in the corresponding function of Platanus. However, it is omitted because of the difficulty in calculating the expected value for the diploid haplotypes, which causes many multiple-hits in mapping of the reads. This function is only applied for the scaffold graph constructed by mapping long-reads, which may cause mis-mappings because of the high sequencing error rate of long-reads.

(2) According to the barcode information of linked-reads

For each edge, the number of linked-reads supporting the common barcode set is counted. Let the numbers corresponding to the edges between the source node and conflicting nodes be b_1 and b_2. If $b_1 > 8 \times b_2$, the edge corresponding to b_2 is removed.

Supplementary Note 4. Divisions of non-bubble nodes in scaffold graph or gapped de Bruijn graph.

To reduce scaffolding of non-bubble contigs consisting of many gaps, nodes that do not contain anchor bubbles are divided according to the criteria described below. Note that gap-rich and non-bubble scaffolds limit the detection of cross-structures (see Methods, Untangling cross structures in the scaffold graph) and the untangling function. For each pair of adjacent contigs (c_1 and c_2) in the node without an anchor bubble, the following two conditions are used to detect divisions.:

(a) c_1 or c_2 has the ($k - 1$)-length overlap to other anchor bubbles, and there is no ($k - 1$)-length overlap between c_1 and c_2;

(b) there is a gap (Ns) between c_1 and c_2 or there is no overlap with length ≤ 20 between c_1 and c_2.

These conditions are applied in the two manners in the different stages of phasing (Supplementary Note 6):
(1) c_1 and c_2 are divided if (a) and (b) are satisfied;
(2) c_1 and c_2 are divided if (a) is satisfied.

Supplementary Note 5. Trimming of sparse edge-regions of nodes in scaffold graph.
This function is designed to reduce sparse scaffolds with a high rate of gaps. For each contig on the node edge, if the length of the adjacent gap is larger than the length of the contig, the node is divided in this gap.

Supplementary Note 6. Iteration of haplotype phasing.
The workflow of the iteration of haplotype phasing is described as the pseudocode detailed below. Note that the specific functions are not executed if the corresponding libraries are not input.

Correspondence between the section-name in this manuscript and functions in the pseudocode:

Construction of a (gapped) de Bruijn graph:
ConstructDeBruijnGraph

Mapping reads to a (gapped) de Bruijn graph:
MapReadsToGraph

Mapping linked-reads to a (gapped) de Bruijn graph:
MapLinkedReadsToGraph

Detection of anchor bubbles for haplotype synteny-based correction:
DetectAnchorBubbles

Untangling cross structures in the (gapped) de Bruijn graph:
(1) UntangleCrossDeBruijnGraphNumberOfLinks
(2) UntangleCrossDeBruijnGraphNumberOfMatchSites
(3) UntangleCrossDeBruijnGraphBarcodes

Construction of the scaffold graph:
ConstructScaffoldGraph

Untangling cross structures in the scaffold graph:
(1) UntangleCrossScaffoldGraphNumberOfLinks
(2) UntangleCrossScaffoldGraphNumberOfMatchSites
(3) UntangleCrossScaffoldGraphBarcodes

Haplotype synteny-based correction:
HaplotypeSyntenyBasedCorrection

Scaffolding to extend each haplotype sequence:
Scaffolding

Divisions of scaffolds in error-candidate positions using mapped read information:
(1) DivideNodesMisAssemblies
(2) DivideNodesSwitchErrors

Removal of low-confidence edges in scaffold graph:
(1) RemoveLowConfidenceEdgeNumberOfLinks
(2) RemoveLowConfidenceEdgeBarcodes

Divisions of non-bubble nodes in scaffold graph or gapped de Bruijn graph:

(1) DivideNonBubbleNodesStrict

(2) DivideNonBubbleNodesLoose

Trimming of sparse edge-regions of nodes in scaffold graph:

TrimSparseEdgeRegionsOfNodes

Input data:

Initial de Bruijn graph resulting from the contig-assembly module: G_{initial}

Short-read paired-end (mate-pair) libraries: L_{short}

Long-read library: L_{long}

Linked-read (barcoded by the 10X-platform): L_{linked}

Definitions:

Graph corresponding to the intermediate result with additional information (such as mapped reads or anchr-bubbles): G

Number of input short-read libraries: m

i-th short-read library: $L_{\text{short}}[i]$ (i is an integer, $1 \leq i \leq m$)

All input libraries including the short-, long-, and linked-read libraries: L_{all}

It is assumed that the short-read libraries are sorted according to the average insert-sizes in ascending order.

<u>Pseudocode:</u>

```
G ← ConstructDeBruijnGraph(G_initial)
G ← DetectAnchorBubbles(G)

for i ← 1 to m
   G ← MapReadsToGraph(G, L_short[i])
end for i
G ← MapReadsToGraph(G, L_long)
G ← MapLinkedReadsToGraph(G, L_linked)

for OuterIterationNumber ← 1 to 4
   G ← ConstructDeBruijnGraph(G)
   for InnerIterationNumber ← 1 to 2
      for i ← to m
         G ← UntangleCrossDeBruijnGraphNumberOfLinks(G, L_short[i])
      end for i
      G ← UntangleCrossDeBruijnGraphNumberOfMatchSites(G, L_long)
      G ← UntangleCrossDeBruijnGraphBarcodes(G, L_long, L_linked)
      G ← UntangleCrossDeBruijnGraphNumberOfLinks(G, L_all)
      G ← UntangleCrossDeBruijnGraphBarcodes(G, L_all)
   end for InnerIterationNumber

   for InnerIterationNumber ← 1 to 2
      for i ← to m
         G ← ConstructScaffoldGraph(G, L_short[i])
         G ← UntangleCrossScaffoldGraphNumberOfLinks(G, L_short[i])
      end for i
      G ← ConstructScaffoldGraph(G, L_long)
      G ← UntangleCrossScaffoldGraphNumberOfMatchSites(G, L_long)
      G ← UntangleCrossScaffoldGraphBarcodes(G, L_long, L_linked)
      G ← UntangleCrossScaffoldGraphNumberOfLinks(G, L_all)
      G ← UntangleCrossScaffoldGraphBarcodes(G, L_all)
```

end for InnerIterationNumber

$G \leftarrow$ DivideNodesSwitchErrors(G, L_{all})
$G \leftarrow$ HaplotypeSyntenyBasedCorrection(G)
$G \leftarrow$ DivideNonBubbleNodesLoose(G)

for InnerIterationNumber $\leftarrow$ 1 to 2
  for $i \leftarrow$ to m
    $G \leftarrow$ ConstructScaffoldGraph(G, $L_{short}[i]$)
    $G \leftarrow$ TrimSparseEdgeRegionsOfNodes(G)
    $G \leftarrow$ UntangleCrossScaffoldGraphNumberOfLinks(G, $L_{short}[i]$)
    $G \leftarrow$ UntangleCrossScaffoldGraphBarcodes(G, $L_{short}[i]$, L_{linked})
    $G \leftarrow$ RemoveLowConfidenceEdgeBarcodes(G, $L_{short}[i]$, L_{linked})
    $G \leftarrow$ Scaffolding(G, $L_{short}[i]$)
  end for i
  if OuterIterationNumber $\leq$ 2
    $G \leftarrow$ DivideNodesMisAssemblies (G, $L_{short}[i]$)
  end if
  else
    $G \leftarrow$ DivideNodesSwitchErrors (G, $L_{short}[i]$)
  end else
end for InnerIterationNumber

$G \leftarrow$ TrimSparseEdgeRegionsOfNodes(G)
$G \leftarrow$ DivideNodesSwitchErrors(G, L_{all})
$G \leftarrow$ HaplotypeSyntenyBasedCorrection(G)

for InnerIterationNumber $\leftarrow$ 1 to 2
  $G \leftarrow$ ConstructScaffoldGraph(G, L_{all})
  $G \leftarrow$ TrimSparseEdgeRegionsOfNodes(G)
  $G \leftarrow$ UntangleCrossScaffoldGraphNumberOfLinks(G, L_{all})
  $G \leftarrow$ UntangleCrossScaffoldGraphBarcodes(G, L_{all})
  $G \leftarrow$ RemoveLowConfidenceEdgeBarcodes(G, L_{all})

```
      G ← Scaffolding(G, L_all)
      if OuterIterationNumber ≤ 2
         G ← DivideNodesMisAssemblies (G, L_all)
      end if
      else
         G ← DivideNodesSwitchErrors (G, L_all)
      end else
   end for InnerIterationNumber

   for InnerIterationNumber ← 1 to 2
      G ← ConstructScaffoldGraph(G, L_long)
      G ← TrimSparseEdgeRegionsOfNodes(G)
      G ← UntangleCrossScaffoldGraphNumberOfLinks(G, L_long)
      G ← UntangleCrossScaffoldGraphBarcodes(G, L_long, L_linked)
      G ← RemoveLowConfidenceEdgeBarcodes(G, L_long, L_linked)
      G ← RemoveLowConfidenceEdgeNumberOfLinks(G, L_long)
      G ← Scaffolding(G, L_long)
      if OuterIterationNumber ≤ 2
         G ← DivideNodesMisAssemblies (G, L_long)
      end if
      else
         G ← DivideNodesSwitchErrors (G, L_long)
      end else
   end for InnerIterationNumber

   G ← TrimSparseEdgeRegionsOfNodes(G)
   G ← DivideNodesSwitchErrors(G, L_all)
   G ← HaplotypeSyntenyBasedCorrection(G)
   G ← DivideNonBubbleNodesStrict(G)
end for OuterIterationNumber
Output(G)
```

Supplementary Note 7. Description of the algorithms of FALCON-Unzip and Supernova.

For FALCON-Unzip, a consensus function followed by re-mapping of reads and variant-calling was implemented[2]. To our knowledge from the original description of Supernova[3], it firstly constructs a de Bruijn graph and the "line" subgraphs which include bubbles. Each "line" corresponds to single locus. Next, it connects "lines" and gaps between them are closed. Finally, it detects linkages between bubbles in the initial de Bruijn graph using barcodes. Since Supernova treated "lines" as units in the intermediate assembly steps, functions such as scaffolding and gap-closing are not used to extend each haplotype independently, in contrast to Platanus-allee.

Supplementary Note 8. Pre-processes of reads.

(1) Paired-ends

Adaptor sequences and low-quality regions in paired-end and mate-pair reads were trimmed using the "platanus_trim" program in Platanus_trim package (version 1.0.7) (http://platanus.bio.titech.ac.jp/pltanus_trim) with default parameters.

(2) Mate-pairs

Adaptor sequences and low-quality regions in paired-end and mate-pair reads were trimmed using the "platanus_internal_trim" program in the Platanus_trim package.

(3) Linked-reads (10X)

For Platanus-allee, using the "longranger basic" command in Long Ranger (version 2.1.2; URL, https://support.10xgenomics.com/genome-exome/software/downloads/latest), barcode sequences were excluded and "BX:Z:" tag information was added to the name-lines in the FASTQ files. For Supernova, the process above was not executed, and raw FASTQ files were input.

(4) Long-reads (PacBio)

Subreads were used for all analyses.

Supplementary Note 9. Estimation of the genome size from short-reads.

The haploid genome size was estimated using an in-house script. For the distribution of the number of 32-mer occurrences generated from Platanus-allee ("out_32merFrq.tsv"), the number of occurrences corresponding to a homozygous peak was detected, with

genome-size = the-total-number-of-32-mers/homozygous-peak-occurrences (5).

Here, 32-mers whose occurrences were small (<number of occurrences corresponding to the bottom between zero and heterozygous peak) were excluded from the calculation to avoid effects from sequencing errors.

Supplementary Note 10. Execution of Platanus-allee.

Platanus-allee (version 2.0.2) was executed using default parameters; no options were specified, except for those for input files and multi-threading. Specifically, the three commands Platanus-allee, "assemble", "phase", and "consensus" were executed, which corresponded to the contig-assembly, phasing, and consensus-scaffolding modules, respectively. The inputs of the "assemble" command were the Illumina paired-ends, not including mate-pairs, linked-reads, or long-reads. All libraries were input to the "phase" and "consensus" commands.

Supplementary Note 11. Execution of Supernova.

Supernova[3] only accepts 10X linked-reads libraries, which were input without other types of data. For each sample, Supernova (version 2.0.0) was executed in two ways, down-sampled-input and full-input. The result showing larger scaffold-NG50 of phased blocks (megabubbles) was selected. The procedures are as follows:

(1) Down-sampled-input

Option for down-sampling, "--maxreads", which determines the number of reads used, was specified for the "supernova run" command. The value for this option was determined as 56 × estimated-genome-size/average-read-length according to the manual (https://support.10xgenomics.com/de-novo-assembly/software/pipelines/latest/using/run

ning). A value of "56" is the optimum coverage depth for Supernova. The average read-length was calculated for pre-processed reads of which barcode-regions were excluded using the "longranger basic" command of Long Ranger (version 2.1.2; URL, https://support.10xgenomics.com/genome-exome/software/downloads/latest). The specific values for the "--maxreads" option are 79292035 and 128849558 for *P. polytes* and *B. japonicum*, respectively.

(2) Full-input

The "supernova run" command was used with the default parameters.

Finally, the results of the down-sampled-input and full-input were selected for *Papilio polytes* and *Bradyrhizobium japonicum*, respectively.

Supplementary Note 12. Execution of FALCON-Unzip.

FALCON-Unzip[2] was executed for subreads of the PacBio long-read libraries as the pipeline consisting of FALCON assembler, unzipping module, and polishing modules (Quiver or Arrow). The version is binary from 11/02/2017, which was applied in the previous study[4]. The binary executable files were downloaded from (https://downloads.pacbcloud.com/public/falcon/falcon-2017.11.02-16.04-py2.7-ucs2.tar.gz).

For each sample, the four parameter sets were tried and the result indicating the maximum primary-contig-NG50 was selected (**Supplementary Table 6**). The descriptions of the parameter sets are as follows:

(1) FALCON-integrate-based

This was used in the previous manuscript and based on the example config file included in the FALCON-integrate package (https://github.com/PacificBiosciences/FALCON-integrate). The parameters modified from the base file, except for parallelization (the number of threads and so on), were as follows:

(i) -s of "pa_HPCdaligner_option" and "ovlp_HPCdaligner_option" (1000 → 100)

This was modified to avoid failures of processes, according to the suggestion from the developer in the issue-page (https://github.com/PacificBiosciences/FALCON/issues/444).

(ii) -t of "ovlp_HPCdaligner_option" (32 → 16)

This was modified to reduce memory-usage and avoid failures. The value was determined according to the instruction in the README of DALIGNER (https://github.com/thegenemyers/DALIGNER) and other example config files in the web page of FALCON (https://pb-falcon.readthedocs.io/en/latest/parameters.html#parameters).

(iii) skip_checks = True

This was added to avoid failures related to the file-system according to the comment from the developer in the issue-page (https://github.com/PacificBiosciences/FALCON/issues/451).

(2) Chin et al. 2016-based

This is the parameter set used in the original paper of FALCON-Unzip[2], which the reviewer suggested. The read-length cutoff is 4 kbp.

(3) Koren et al. 2018-based

This was used in the previous study[4] to assemble the human NA12878 data. In addition to the original value of read-length cutoff (5 kbp), 4 kbp was also tested.

The part of configuration files of FALCON, excluding the parameters related to genome sizes and a machine-environment (speciation of a job-scheduler, the number of concurrent jobs, and so on), are as follows:

(1) FALCON-integrate-based

Start of the common parameters in a config file

```
input_type = raw
```

length_cutoff = 15000

length_cutoff_pr = 15000

pa_HPCdaligner_option = -v -B128 -t16 -e.70 -l1000 -s100
ovlp_HPCdaligner_option = -v -B128 -t16 -h60 -e.96 -l500 -s100

pa_DBsplit_option = -x500 -s400
ovlp_DBsplit_option = -x500 -s400

falcon_sense_option = --output_multi --min_idt 0.70 --min_cov 4 --max_n_read 200 --n_core 16

overlap_filtering_setting = --max_diff 100 --max_cov 100 --min_cov 1 --bestn 10 --n_core 16

skip_checks = True

End of the common parameters in a config file

(2) Chin et al. 2016-based

Start of the common parameters in a config file

input_type = raw

length_cutoff = 4000

length_cutoff_pr = 4000

pa_HPCdaligner_option = -v -dal128 -e0.75 -M24 -l1800 -k18 -h240 -w8 -s100
ovlp_HPCdaligner_option = -v -dal128 -M24 -k24 -h750 -e.96 -l1500 -s100

pa_DBsplit_option = -a -x500 -s400
ovlp_DBsplit_option = -s400

falcon_sense_option = --output_multi --min_idt 0.70 --min_cov 4 --max_n_read 400 --n_core 8
falcon_sense_skip_contained = False

overlap_filtering_setting = --max_diff 80 --max_cov 120 --min_cov 4 --n_core 24

End of the common parameters in a config file

(3) Koren et al. 2018-based

(i) The version in which length cutoff = 5 kbp

Start of the common parameters in a config file

```
input_type = raw

length_cutoff = 5000

pa_HPCdaligner_option = -v -dal128 -e0.75 -M24 -l1200 -k18 -h256 -w8 -s100
ovlp_HPCdaligner_option = -v -dal128 -M24 -k24 -h600 -e.96 -l1800 -s100
pa_DBsplit_option = -x500 -s400
ovlp_DBsplit_option = -s400

falcon_sense_option = --output_multi --min_idt 0.70 --min_cov 4 --max_n_read 200 --n_core 8

overlap_filtering_setting = --max_diff 120 --max_cov 120 --min_cov 2 --n_core 24
```

End of the common parameters in a config file

(ii) The version in which length cutoff = 4 kbp

Start of the common parameters in a config file

```
input_type = raw

length_cutoff = 4000
```

pa_HPCdaligner_option = -v -dal128 -e0.75 -M24 -l1200 -k18 -h256 -w8 -s100
ovlp_HPCdaligner_option = -v -dal128 -M24 -k24 -h600 -e.96 -l1800 -s100
pa_DBsplit_option = -x500 -s400
ovlp_DBsplit_option = -s400

falcon_sense_option = --output_multi --min_idt 0.70 --min_cov 4 --max_n_read 200 --n_core 8

overlap_filtering_setting = --max_diff 120 --max_cov 120 --min_cov 2 --n_core 24

End of the common parameters in a config file

Note that the parameters specific to our machine-environment, such as the file locations or number of cores, are omitted above. The parameter that was varied for the samples was the estimated genome size ("genome_size"). The specific values were as follows:

C. elegans: 100000000

P. polytes: 233000000

B. japonicum: 386000000

H. sapens: 2900000000

For the "unzip" module of FALCON-Unzip, only parameters related to the machine-environment (speciation of a job-scheduler, the number of concurrent jobs, and so on) were specified.

Note that the result of the Koren et al. 2018-based parameter set for the human sample was downloaded from the web page related to the original study (https://gembox.cbcb.umd.edu/triobinning/)[4].

After the unzipping (phasing) procedure, each read was distributed to the contig using "fc_quiver" in the unzipping module. Due to execution errors for the polishing step of fc_quiver, distributed reads were re-aligned by "pbalign" aligner and the polishing tool (Quiver or Arrow) was executed as the in-house script. The pbalign, Quiver, and Arrow tools are components of SMRT link (version 3.1.1.182868; URL,

https://www.pacb.com/support/software-downloads). Quiver was applied for *P. polytes* and *B. japonicum* data, while Arrow was applied for *C. elegans* data, considering the difference in the sequencing platforms and compatibilities of the file formats.

Supplementary Note 13. Division of the primary contigs of FALCON-Unzip to make the phased block set.

The primary contigs of FALCON-Unzip have mosaic-structures of haplotypes and were divided after the boundaries of the blocks were determined by aligning haplotigs, which are non-mosaic haplotype sequences used as benchmarks of phasing performance. The procedures were as follows:

(1) Haplotigs were aligned as queries to the primary contigs by Minimap2[5] (version 2.0-191) with the options of "-c -k 19 -p 0".

(2) Local alignments in the raw results of Minimap2 (PAF format) whose sequence-identity ≥ 0.8 and alignment-length ≥ 1000 were extracted.

(3) From the set of filtered local alignments, each alignment between the associated pair of contigs was extracted. The associations of contigs were determined according to the information FALCON-Unzip output. Specifically, a pair of contigs is associated if the prefixes of the sequence names (strings in the left sides of "_") are identical.

(4) For each haplotig, the left-most and right-most local alignments were determined for the coordinate on the haplotig. If multiple local alignments had identical positions, those used for the downstream step were selected according the number of match-sites (larger alignment was selected). If the left-most and right-most alignments indicated a different strand, the local alignment with the largest number of match-sites was selected.

(5) For each haplotig, the corresponding region in the primary contig was determined according to the positions of alignments selected in step (4). The primary contigs were divided at the boundaries of these regions. Finally, the divided primary contigs and haplotigs were combined into a single FASTA file, and contigs with lengths ≤ 500 were discarded.

Supplementary Note 14. Execution of Pilon.

The procedures for Pilon[6] version 1.22 were as follows:

(1) PE reads were mapped to the contig set of FALCON-Unzip using BWA-MEM (version 0.7.12-r1044) [7] with the default parameters ("index" and "mem" commands) of the paired-end mode. This contig set includes the primary-contigs and haplotigs that had been polished using PacBio reads. Input PEs were common to those for Platanus-allee, which typically had 80× coverage depth.

(2) The BAM file of mapping results was sorted according to the positions by the "sort" command of SAMtools (version 1.3.1)[8].

(3) Pilon was executed using the sorted BAM file with the default parameters, except for that of multi-threading (-t). For the human NA12878 sample only, input contigs (FASTA format) and the sorted BAM file were split using the in-house script and Pilon were further parallelized. This procedure also reduced memory usage.

Supplementary Note 15. Execution of Purge-Haplotigs.

The procedures for Purge-Haplotigs[9] version 17SEP2018 were as follows:

(1) The input contigs were those of FALCON-Unzip that were polished by Pilon (**Supplementary Note 14**). The PacBio reads were re-mapped to these contigs using Minimap2 (version 2.0-r191) with the option of "-ax map-pb".

(2) The output of Minimap2 was converted to the position-sorted BAM file using SAMtools[8].

(3) Inputting the BAMfile and contigs, the "purge_haplotis readhist" command was executed to generate the histogram of coverage depths.

(4) The thresholds related to coverage depths (-l, -m and -h) were determined manually using the histogram made in (3), and "purge_haplotigs contigcov" was executed inputting the coverage depth information (output of (3)).

(5) To generate the curated contigs, the "purge_haplotigs purge" command was executed inputting the output of (4) and contigs. The final phased block was constructed as the similar procedures described in **Supplementary Note 13**.

Supplementary Note 16. Execution of GenomeScope.

The result was shown in **Supplementary Table 5**. The version was 1.0. For the pre-processed (trimmed) Illumina PE, Jellyfish[10] (version 2.2.3), a *k*-mer counter, was applied with the options of " -m 21 -C" and "-h 1000000000" for "count" and "histo" commands, respectively. The *k* value, 21, was determined according to the recommendation of the manual. GenomeScope[11] was executed as the R script inputting the result of Jellyfish (histogram of 21-mer occurrences) and the mean read length.

Supplementary Note 17. Counting exactly matched mate-pairs for phased blocks in the *P. polytes* benchmark.

The results are included in **Table 1**. For each tool, the reads of mate-pairs (nominal insert-size, 15 kbp) were mapped to phased blocks using BWA-MEM[7] (version 0.7.12-r1044; "index" and "mem" commands) with the default parameters as the single-end reads. Here, forward and reverse files were separately input into the "mem" command without the "-p" option. For the two BAM files of mapped forward and reverse reads, the number of pairs in which both reads mapped to the same sequence and indicated exact matches (edit-distance = 0 and end-to-end alignment) were determined using the in-house script. Note that this analysis was performed only for *P. polytes* (butterfly), which did not have the reference genome or Moleculo contigs.

Supplementary Note 18. Calculation of the numbers of switch-errors and mis-assemblies for the *C. elegans* data.

The schematic model is shown in **Supplementary Fig. 5**. The result is shown in **Supplementary Table 13**. The procedures were as follows:

(1) The scaffolds (contigs) were divided into non-overlapping and fixed-length (1 kbp or 10 kbp) fragments. Fragments with gap rates ('N's) ≥50% were excluded.

(2) Fragments from the scaffolds were aligned to the reference sequences consisting of the genomes of the two strains, N2[12] and CB4856[13], using Minimap2[5] (version 2.7-r664) with the options of "-c -p 1 -k 19". Additionally, alignments were extracted and used in the steps below if the following condition was satisfied:

$$\text{alignment-coverage} \geq 0.9 \text{ and sequence-identity} \geq 0.95 \quad (6)$$

Sequence-identity was calculated based on a value of the "dv" tag (approximate per-base sequence-divergence) in a PAF file (output of Minimap2) such that sequence-identity = 1 − dv-value.
Note that the total length of the aligned fragments and sequence identity in **Supplementary Table 13** were calculated based on the extracted alignments above.

(3) Fragments with multiple best-hits for alignment-scores (values of "AS" tag) were excluded.

(4) For each pair of adjacent unique-hit fragments in a scaffold, a switch-error was detected if the pair was aligned to homologous chromosomes. For this, let the distance between the aligned positions of the pair on the same chromosome be d_{align} and distance between the positions of pair on the scaffold be d_{scaffold}. A mis-assembly was detected if either of two conditions was satisfied:

The pair was aligned to different non-homologous chromosomes

or

$$| d_{\text{align}} - d_{\text{scaffold}} | > \max(d_{\text{align}}, d_{\text{scaffold}})/2 \quad (7)$$

Supplementary Note 19. Calculation of the numbers of mis-assemblies in consensus sequences for the *C. elegans* data.

The result is shown in **Supplementary Table 14**. The procedures are similar to that described in **Supplementary Note 18**, except for the following points:

(1) The target of the evaluation is consensus sequences, not phased blocks.
(2) The reference genome for the evaluation is that of *C. elegans* N2 strain.
(3) Counting of the number of switch-errors is omitted.

Supplementary Note 20. Depiction of dot plots of sequence alignments.

This procedure corresponds to **Fig. 2a and 4d**. First, the target pair of sequences were divided into non-overlapping fixed-length (1 kbp) fragments and stored as two FASTA files. Next, to perform alignment, the two files were input into the "nucmer" command in the MUMmer package[14] (version 3.1) with the options of "-maxmatch -nosimplify". Finally, the resulting "delta" file was input into the "mummerplot" command to generate the dot plot. Note that we modified the raw gnuplot script from mummerplot to change the colors, font, and tics.

Supplementary Note 21. Depiction of alignments as lines.

This procedure corresponds to **Fig. 2e, 3b, 4c, and 4d**. First, the targets of two assembly sets were aligned with each other using Minimap2 using the options "-c -k 19 -p 0". Two sequences were associated if one of the sequences indicated the best hit according to the number of match-sites. Note that this process did not impose the bidirectional best hits. Each associated sequence pair was re-aligned in a similar manner to align FALCON-Unzip's haplotigs to its primary contigs (**Supplementary Note 13**). After determining the ranges of positions of the alignments, these ranges were drawn as lines.

Supplementary Note 22. Construction of the phased blocks of the pipeline of Mostovoy *et al.* (2016)[15].

The consensus haploid scaffold (FASTA format) and phasing information (VCF format) were downloaded from http://kwoklab.ucsf.edu/resources/. The "bcftools consensus" command of BCFTools[8] (version 1.2-157-g8deae27) was used to obtain the sequence of heterozygous phased blocks in FASTA format. Additionally, the consensus haploid scaffolds were divided at the boundary of the phased blocks, and the regions not assigned to a haplotypes (unphased) were converted into the FASTA format using an in-house script. The resulting phased block had a similar format to those of Platanus-allee and Supernova, enabling comparisons.

Supplementary Note 23. Evaluation of phased blocks of the human NA12878 using the Platinum variant set.

We performed the evaluation using the phased variant set of NA12878, called Platinum set, previously derived from the trio data[16]. Note that the variants were basically detected on the basis of mapping of short-reads and possibly HDRs were not reflected, but this evaluation was valuable as the one not depending on the Moleculo data. For Recalls and precisions measured based on fixed-length fragments from the haplotypes of the Platinum set (**Supplementary Table 16**), the trend was consistent with those for the Moleculo contigs; (1) Supernova achieved the highest recalls, (2) Platanus-allee achieved the highest precisions and (3) Platanus-allee with PacBio or 10X exceeded FALCON-Unzip (Pilon, PH) both for recalls and precisions. For the rate of switch-errors and mis-assemblies (**Supplementary Table 17**), FALCON-Unzip and Supernova recorded the lowest error-rate (# (switch + mis-assemblies)/fragment), and the ranking varied depending on the fragment size for evaluations. Data (10X) are effective to reduce the rate of switch-errors of Platanus-allee, resulting in the comparable performances to the others.

The specific procedures were as follows:

(1) The VCF file of the Platinum set and the corresponding reference genome (hg38) were downloaded from ftp://platgene_ro@ussd-ftp.illumina.com/2017-1.0/hg38/small_variants/NA12878/NA12878.vcf.gz and http://hgdownload.cse.ucsc.edu/goldenPath/hg38/bigZips/hg38.fa.gz .

(2) The VCF file was compressed using the "bgzip" tool (version 1.3.1) and indexed using "tabix" tool (version 1.3.1). These tools are related to the SAMtools[8].

(3) The sequences of two haplotypes were constructed using the "consensus" command of BCFTools[8].

(4) Sequences not targeted in the Platinum set (Y chromosome and unlocalized sequences) were excluded.

(5) The constructed Platinum haplotype sequences and the benchmarked phased blocks were divided into 1k or 5k-fragments (non-overlapping) and used to measure recalls and precisions of the phased blocks using an approach similar to that used when analyzing *C. elegans*. The indicators were defined as follows: recall, number of Platinum-fragments matched to phased blocks/number of all Platinum-fragments; precision and number of phased block-fragments matched to Platinum haplotype/number of all phased block fragments.

(6) Using the constructed Platinum haplotypes as the reference sequences, the number of switch-errors and mis-assemblies were counted similar to the *C. elegans* benchmark (**Supplementary Note 18**)

Supplementary Note 24. Evaluation of the MHC region of the human NA12878 using the previous typing result.

Specifically, CDS and mRNA sequences of the loci in the IPD-IMG/HLA database were aligned to phased blocks, and a typing result as diploid were determined for each locus.

The results are included in **Supplementary Table 18**. In contrast to the genome-wide benchmarks, the performances of Supernova and Mostovoy *et al*. 2016 pipeline were worse compared with the others, and it is inferred that these tools cannot phase highly divergent regions accurately. As the advantage of the *de novo* haplotyping of MHC regions, new haplotype sequences absent in the database can be obtained.

For each benchmarked tool, the concordance between its phased blocks and the previous typing result[17] were checked. The six targeted loci and the haplotypes of NA12878 previously inferred were as follows (locus-name, haplotype):

HLA-A, 01:01/11:01
HLA-B, 08:01/56:01
HLA-C, 01:02/07:01
HLA-DQA1, 01:01/05:01
HLA-DQB1, 02:01/05:01
HLA-DRB1, 01:01/03:01

The checking procedures were as follows:

(1) The CDS and mRNA sequences of the MHC (HLA) loci were downloaded from the IPD-IMGT/HLA database (release date, 18/10/2018). The URLs were:
CDS, ftp://ftp.ebi.ac.uk/pub/databases/ipd/imgt/hla/hla_gen.fasta
mRNA, ftp://ftp.ebi.ac.uk/pub/databases/ipd/imgt/hla/hla_nuc.fasta

(2) The null-allele sequences (alleles of no expression) were excluded from the downloaded CDS and mRNA sets.

For each locus, the steps (3)–(7) were applied. The procedures below were performed separately for CDS or mRNA.

(3) The sequences in database were aligned as queries to the phased blocks using Minimap2 (version 2.7-r664) with the option of "-x splice".

(4) The alignments whose identity ≥90% and query-coverage ≥50% were extracted.

(5) For each scaffold, the alignment whose edit-distance (value of "NM" tag in PAF file) was minimum was selected.

(6) Among the all alignments selected in the step (5), the two alignments whose edit-distances were minimum were further extracted.

(7) For the two alignments extracted in the step (6), if these (i) corresponded to a paired phased block (bubble) and (ii) the types of selected database sequences matched the ones of previous study, the locus was counted as "correct typing".

(8) For all correctly typed loci, the number that indicated exact-match (edit-distance = 0 for both alleles) were also counted.

Supplementary Note 25. Search of HLA-related genes for the human NA12878 phased blocks.

The targeted genes were *HLA-DRB5* and *HLA-DRB3*. For each gene, CDSs from all isoforms were aligned to phased blocks using Minimap2[5] (version 2.7-r664) with the option "-c -x splice". If an alignment with identity ≥90% and query-coverage ≥50% was detected by the in-house script, the gene was counted as "found".

Supplementary Note 26. Benchmarking for the other organisms using the public data.

First, we performed the benchmark for *A. thaliana* F1-hybrid[2] (**Supplementary Table 23–25**) as the alternative sample whose parental genomes were available. The estimated heterozygosity was 1.05% (**Supplementary Table 5**), which was the middle value between the highly heterozygous samples (*P. polytes* and *B. japonicum*) and lowly heterozygous ones (*C. elegans* and *H. sapiens*) in this study. Here, utilizing the assemblies of the parental strains as reference genomes, recalls and precisions were measured. Probably because MP libraries were not available, the performances of Platanus-allee were generally poor, which is consistent with the benchmarks of the other samples. FALCON-Unzip outperformed Platanus-allee for all indicators about phased blocks except for precision of 1k-mers (**Supplementary Table 23, 25**).

Next, the heterozygous (estimated value, 2.97%) plant sample with MP libraries, *Prunus yedonensis* (cherry blossom)[18], was used for benchmark. Note that the entire data of this sample was too rich compared with the other samples in this study, PE was downsampled to 80× and the number of MPs were limited to four (insert sizes, ≤15 kbp). In contrast to *A. thaliana* F1-hybrid, Platanus-allee indicated the high performances compared to the FALCON-based public assembly (**Supplementary Table 23**), supporting the effectiveness of MP libraries.

Finally, as the alternative mammal, *Pteropus alecto* (bat)[19], was tested. This sample was highly heterozygous (estimated value, 0.99%) relative to the human. As a result only from the PE and 4 MPs, Platanus-allee scaffold-NG50 of phased blocks reached 342 kbp, which was much higher than 4.09 kbp of Platanus-allee and 109 kbp of FALCON-Unzip for the human sample. This supports the hypothesis that the low heterozygosity (≤0.1%) makes Platanus-allee's phased blocks short and it can achieve long ones for highly heterozygous organisms with the genome size >2 Gbp.

Contig-NG50 of phased blocks of FALCON-Unzip in the case of *A. thaliana* (~3 Mbp) is exceptionally large among the other species targeted in this study (maximum, 413 kbp; **Table 1, Supplementary Table 23**). Even for the other species in the original study of FALCON-Unzip, *Vitis vinifera* (grape; estimated size, 500 Mbp) and *Clavicorona pyxidata* (fungus; estimated size, 42 Mbp), contig-NG50s of phased blocks of FALCON-Unzip were just 736 kbp and 464 kbp, respectively (results were downloaded and evaluated in the same way for the other species), and we suspected that the high performance of FALCON-Unzip for the *A. thaliana* F1-hybrid is the rare case. In summary, these benchmarks confirm the tendency of Platanus-allee about input libraries and illustrates the fluctuation of the FALCON-Unzip performances.

Supplementary Note 27. Benchmarking for fully simulated heterozygous data.

To systematically investigate the effect of heterozygosity, we prepared data sets including all types of libraries fully simulated *in silico*. The used simulators of reads were ART[20], LongISLND[21] and LRSIM[22] for PE (MP), PacBio and 10X, respectively. The target species was *C. elegans* (N2) and the configuration of the libraries was similar to that in the benchmark of synthetic diploid *C. elegans* (N2 and CB4856). Heterozygous SNV, indels and structural variants were simulated by pIRS[23], and the heterozygosities were simulated as 0.1–2%.

The results are shown in **Supplementary Table 26 and 27**. The target organism was *C. elegans* N2, and the library set (PE, 3 MPs, 10X, and PacBio) were simulated as the following procedures (1)–(4). Matching of the types, mean read-lengths, and total lengths of the libraries to those of the actual data of *C. elegans* synthetic diploid were attempted (**Supplementary Table 1**). The simulated reads were pre-processed as the similar way for the actual data (**Supplementary Note 8**).

(1) Generation of the alternative reference haplotype.

The simulator was pIRS[23] (version 110) and the base reference genome was the nuclear chromosomes of *C. elegans* N2 (accession numbers NC_003279.8, NC_003280.10, NC_003281.10, NC_003282.8, NC_003283.11, NC_003284.9). The simulated heterozygosities were 0.1%, 0.5%, 1.0%, and 2.0%. For each value of heterozygosity, an alternative reference genome was simulated using the "pirs diploid" command with the following parameters:

SNP rate (-s): heterozygosity (converted from percentage to per-base rate)
Indel rate (-d): 1/10 of SNP rate
Structural variation rate (-v): 1/100 of SNP rate

The base reference genome and the generated alternative genome were concatenated and input into the simulations of reads (2)–(4).

(2) Illumina PE and MP simulation

The simulator was ART[20] (version 2.5.8), and five libraries (1 PE and 4 MPs) were generated. The options of coverage depth (-f), read length, (-l), mean insert size (-m) and standard deviation of insert size (-s) were described below:

PE: -f 40 -l 250 -m 600 -s 60
MP1: -f 20 -l 100 -m 5000 -s 500
MP2: -f 20 -l 100 -m 9000 -s 900
MP3: -f 20 -l 100 -m 12000 -s 1200
MP4: -f 20 -l 100 -m 16000 -s 1600

For the PE, since the error profile of HiSeq2500 with read length 250 bp was not included in the ART package, the corresponding one was generated using "art_profiler_illumina profile" command inputting the actual reads of the *C. elegans* N2 strain sequenced in this study, and the resultant profile was used by ART. For the MPs, the prepared profile HiSeq2500 with read length 100 bp (-ss HS25) was used.

(3) PacBio simulation

The simulator was LongISLND[21] (version 0.9.5). To our knowledge, this is the only simulator that can generate files acceptable to FALCON, which includes movie ID and other information.

Reads were simulated utilizing "simulate.py" in the LongISLND package with the option to ouput PacBio-BAM files (--read_type clrbam). The coverage depth for diploid genome was set to 100× (--coverage 100), which was 200× for the haploid genome. The resulting BAM files were converted to FASTA files using the "samtools fasta" command of SAMtools[8]. Finally, the read-group information in the FASTA files were modified for "fc_quiver" in FALCON-Unzip using the in-house script, referring to the

document provided by Pacific Biosciences (https://pacbiofileformats.readthedocs.io/en/3.0/BAM.html).

(4) 10X linked read simulation

The simulator was LRSim[22] (version 1.0). Default parameters, such as total read length and the number of partitions (barcodes), were adjusted for the human genome (haploid size ~3 Gbp), and the parameters were scaled to the *C. elegans* genome (haploid size ~0.1 Gbp). The script used was "simulateLinkedReads.pl" in the LRSIM package and the option was "-p sim -z 12 -o -x 20 -f 50 -t 50 -m 10".

Supplementary Note 28. Calculation of precision and recall for the *Arabidopsis thaliana* F1-hybrid benchmark.

The result is shown in **Supplementary Table 25**. The procedures were similar to those used in the benchmark of *C. elegans* synthetic diploid data, but the assemblies of parental strains downloaded were used as the reference genome. The assemblies of the parental strains and the phased blocks benchmarked were divided into 1k or 5k-fragments (non-overlapping) and used to measure recalls and precisions. The indicators were defined as follows: recall, number of parental-assembly-fragments matched to phased blocks/number of all parental-assembly-fragments; precision and number of phased block-fragments matched to parental assembly/number of all phased block fragments.

Supplementary Note 29. Measurement of run time of Platanus-allee.

The results are shown in **Supplementary Table 9 and 10**. For the machine environment, the number of CPUs were 20, the model name of CPU was Intel(R) Xeon(R) CPU E5-2687W v3, the clock rate of CPP was 3.10 GHz, and the RAM amount was 784 Gb. Platanus-allee was executed with the option specifying the number of threads was 20 (-t 20), and the times (real and CPU time) were measured using "time" command.

Supplementary Note 30. Descriptions of the costs of sequencing technologies.

(1) PE

The cost is minimum. It has a variety of uses that include all modules of Platanus-allee and even for polishing of PacBio-based contigs. Using *k*-mer analysis, preliminary information of a genome such as size, heterozygosity and repetitiveness, can be derived. Therefore, it may be considered that PE is first sequenced to determine an entire plan of a genome project.

(2) MP

The sequencing cost is common to PE, but it consists of additional costs to library preparations including kits and labor. Note that multiple MPs (*e.g.* 3 MPs, coverage depth 40 for each) would not lead to a higher cost compared with PacBio having the same total coverage depth.

(3) PacBio

The sequencing cost is several-fold higher than that of the short-reads (Illumina). Run time is short (<1 day) and experimental steps are fewer than that for multiple MPs, possibly reducing the labor cost.

(4) 10X

The sequencing cost is common to PE, but it consists of additional costs for library preparation. Remarkably, Supernova achieved the high performance for the human sample using only this library. For heterozygous samples, such as humans, the combination of this library and Supernova might be the most cost-effective strategy, if HDRs and repeat-rich regions are not targeted.

Supplementary Note 31. Previous public data used in this study.

(1) *C. elegans* reference genome[12,13]

The GenBank assembly accession of the N2 and CB4856 strains are GCA_000002985.3 [https://www.ncbi.nlm.nih.gov/assembly/GCF_000002985.6] and GCA_000975215.1 [https://www.ncbi.nlm.nih.gov/assembly/GCA_000975215.1/], respectively. The accession numbers of the reference chromosomes (I–V and X) were as follows:

N2 strain:

NC_003279.8, NC_003280.10, NC_003281.10, NC_003282.8, NC_003283.11, NC_003284.9

CB4856 strain:

CM003206.1, CM003207.1, CM003208.1, CM003209.1, CM003210.1, CM003211.1

(2) PE and MP libraries of the human NA12878[24]

Raw reads were downloaded from the SRA database and had the following accession numbers:

PE (insert size 470): SRR891258, SRR891259
[https://www.ncbi.nlm.nih.gov/sra/SRR891258]

MP (insert size, 2k): ERR262997
[https://www.ncbi.nlm.nih.gov/sra/ERR262997]

(3) PE library of the human NA12878 to estimate heterozygosity[16]

Raw reads were downloaded from the SRA database. The accession numbers are ERR174330–ERR174339.

(4) PacBio reads (P6-C4) of the human NA12878 PacBio long-reads

Raw reads deposited under BioProject PRJNA323611 [https://www.ncbi.nlm.nih.gov/bioproject/PRJNA323611]were downloaded from the SRA database. Their accession numbers are SRR3657588–SRR3658515. The files were downloaded as the compressed HDF5 format and converted to the FASTA format for the benchmarked tools.

(5) Moleculo contigs of the human NA12878[25]

The data were downloaded from the FTP site of the 1000 Genome Project [http://ftp.1000genomes.ebi.ac.uk/vol1/ftp/phase3/integrated_sv_map/supporting/NA12878/moleculo] as files: NA12878_moleculo_1.tar, NA12878_moleculo_2.tar and NA12878_moleculo_3.tar.

(6) FALCON-Unzip assembly of the human NA12878[4]

The FALCON-Unzip assembly of the Koren et al. 2018-based parameter set for the human sample was downloaded from the web page related to the original study[3]. The URLs are below:
[https://gembox.cbcb.umd.edu/triobinning/hsapiens_falcon_primary.fasta.gz]
[https://gembox.cbcb.umd.edu/triobinning/hsapiens_falcon_alts.fasta.gz]

(7) 10X linked-reads and Supernova assembly of the human NA12878[2]

The barcoded FASTQ file and FASTA files of the Supernova (version 2.0.0) assembly were downloaded from the 10X Genomics website [https://support.10xgenomics.com/de-novo-assembly/datasets/].

(8) Human NA12878 assembly and phasing result of the pipeline of Mostovoy *et al.* (2016)[15]

The assembly (FASTA) and the phasing (VCF) data were downloaded from the Kwok Laboratory website, University of California, San Francisco [http://kwoklab.ucsf.edu/resources/].

(9) PE and PacBio libraries of *A. thaliana* F1-hybrid[2]

Raw reads were downloaded from the SRA database and had the following accession numbers:

PE (insert size, 400): SRR3703081, SRR3703082, SRR3703105

PacBio: SRR3405292, SRR3405294, SRR3405296, SRR3405301, SRR3405302, SRR3405303, SRR3405304, SRR3405305, SRR3405306, SRR3405307, SRR3405308, SRR3405309, SRR3405310

The files were downloaded as the compressed HDF5 format and converted to the FASTA format for the tools benchmarked.

(10) Public assembly of *A. thaliana* F1-hybrid[2]

The data sets related to the previous study[2] was downloaded from [https://downloads.pacbcloud.com/public/dataset/PhasedDiploidAsmPaperData/FUNZIP-PhasedDiploidAssemblies.tgz] as compressed archive file (tgz). The FALCON-Unzip assembly of the F1-hybrid was extracted as the file under "cvi-0_col-0_f1_assembly" directory. The assemblies of the parental strains were also used for the evaluation, and the corresponding directory names were "col-0_assembly" and "cvi-0_assembly".

(11) PE, MP and PacBio libraries of *Prunis yedonensis* (cherry blossom)[18]

The corresponding BioProject ID is PRJNA382466 [https://www.ncbi.nlm.nih.gov/bioproject/382466]. Raw reads were downloaded from the SRA database and had the following accession numbers:

PE (insert size, 250): SRR6957274
PE (insert size, 500): SRR6950931
MP (insert size, 5k): SRR6987272
MP (insert size, 3k): SRR6981898
MP (insert size, 10k): SRR7026335
MP (insert size, 15k): SRR7027861
PacBio: SRR6928153

(12) Public assembly of *P. yedonensis*[18]

The FALCON-based assembly, Pyn_1.0, was downloaded from the NCBI GenBank database. The GenBank accession number is GCA_002966975.1 [https://www.ncbi.nlm.nih.gov/assembly/GCA_002966975.1/].

(13) PE and MP libraries of *Pteropus alecto* (bat)[19]

The corresponding BioProject ID is PRJNA171993 [https://www.ncbi.nlm.nih.gov/bioproject/171993]. Raw reads were downloaded from the SRA database and had the following accession numbers:

PE (insert size, 250): SRR534483, SRR534484, SRR534485
PE (insert size, 800): SRR534540
MP (insert size, 2k): SRR534545, SRR534546
MP (insert size, 5k): SRR534541, SRR534542
MP (insert size, 10k): SRR534543, SRR534544
MP (insert size, 20k): SRR534547, SRR534548

(14) Public assembly of *P. alecto*[19]

The SOAPdenovo-based assembly, ASM32557v1, was downloaded from the NCBI RefSeq database. The RefSeq accession number is GCF_000325575.1 [https://www.ncbi.nlm.nih.gov/assembly/GCF_000325575.1/] .

Supplementary Tables

Supplementary Table 1. Sequencing reads of *P. polytes* (swallowtail butterfly).

Status	Data generation	Library type	Nominal insert length (bp)	Mean read length (bp)	Total length (bp)	Expected sequence coverage (×)
Raw	This study	PE	600	250	18,422,770,000	77
		MP	2,000	100	12,429,084,800	52
		MP	4,500	100	13,282,216,200	55
		MP	9,000	100	13,056,832,200	54
		MP	15,000	100	14,161,245,000	59
		10X	400	181	63,807,575,240	266
		PacBio		11,839	23,768,889,877	99
Pre-processed	This study	PE	600	244	17,965,038,466	75
		MP	2,000	88	8,102,416,469	34
		MP	4,500	88	8,870,930,197	37
		MP	9,000	88	8,411,535,686	35
		MP	15,000	89	9,611,841,863	40
		10X	400	170	59,753,502,780	249
		PacBio		11,839	23,768,889,877	99
Platanus-allee input (downsampled)	This study	PE	600	244	17,965,038,466	75
		MP	2,000	88	8,102,416,469	34
		MP	4,500	88	8,870,930,197	37
		MP	9,000	88	8,411,535,686	35
		MP	15,000	89	9,611,841,863	40
		10X	400	170	59,753,502,780	249
		PacBio		11,834	4,797,848,267	20

Supplementary Table 2. Sequencing reads of *B. japonicum* (amphioxus).

Description	Data generation	Library type	Nominal insert length (bp)	Mean read length (bp)	Total length (bp)	Expected sequence coverage (×)
Raw	This study	PE	400	150	52,414,195,500	134
		MP	3,000	150	59,746,723,200	153
		MP	5,000	150	61,589,025,600	158
		MP	10,000	150	64,166,631,000	165
		10X	400	181	47,871,050,864	123
		PacBio		11,083	60,720,426,756	156
Pre-processed	This study	PE	400	141	47,878,995,913	123
		MP	3,000	125	40,958,215,785	105
		MP	5,000	126	42,321,667,683	109
		MP	10,000	126	44,390,107,683	114
		10X	400	170	44,829,520,008	115
		PacBio		11,083	60,720,426,756	156
Platanus-allee input (downsampled)	This study	PE	400	141	31,200,055,276	80
		MP	3,000	125	15,599,883,548	40
		MP	5,000	126	15,599,561,680	40
		MP	10,000	126	15,600,169,674	40
		10X	400	170	44,829,520,008	115
		PacBio		11,081	7,799,028,962	20
Synthetic long reads for evaluation	This study	Moleculo		5,082	5,273,561,298	14

Supplementary Table 3. Sequencing reads of *C. elegans* (N2 and CB4856 strains).

Description	Data generation	Library type	Nominal insert length (bp)	Mean read length (bp)	Total length (bp)	Expected sequence coverage (×)
N2 raw	This study	PE	600	250	19,130,905,500	191
		MP	5,000	100	5,815,877,600	58
		MP	9,000	100	5,145,968,200	51
		MP	12,000	100	6,826,349,800	68
		MP	16,000	100	4,961,450,000	50
		PacBio		11,098	9,627,614,552	96
CB4856 raw	This study	PE	600	250	13,632,853,000	136
		MP	5,000	100	5,148,965,600	51
		MP	9,000	100	6,840,699,000	68
		MP	12,000	100	4,398,050,600	44
		MP	16,000	100	6,036,177,800	60
		PacBio		9,919	12,033,513,335	120
N2 pre-processed	This study	PE	600	234	17,930,658,195	179
		MP	5,000	86	3,800,705,916	38
		MP	9,000	87	3,482,139,707	35
		MP	12,000	86	4,155,802,409	42
		MP	16,000	88	3,181,219,126	32
		PacBio		11,098	9,627,614,552	96
CB4856 pre-processed	This study	PE	600	233	12,710,445,617	127
		MP	5,000	87	3,444,405,510	34
		MP	9,000	86	4,495,618,182	45
		MP	12,000	87	2,876,490,834	29
		MP	16,000	86	3,775,913,883	38
		PacBio		9,919	12,033,513,335	120
Mixed Platanus-allee input (downsampled)	This study	PE	600	234	7,999,920,896	80
		MP	5,000	86	3,999,911,383	40
		MP	9,000	87	3,999,938,593	40
		MP	12,000	87	3,999,967,578	40
		MP	16,000	87	4,000,089,881	40
		PacBio		10,473	2,005,270,264	20
Mixed FALCON-Unzip input (downsampled)	This study	PacBio		10,482	8,028,062,739	80
		PacBio		10,479	12,037,971,187	120
		PacBio		10,485	16,059,527,455	161
		PacBio		10,479	19,260,453,715	192

Supplementary Table 4. Sequencing reads of *H. sapiens* (human) NA12878 .

Status	Data generation	Library type	Nominal insert length (bp)	Mean read length (bp)	Total length (bp)	Expected sequence coverage (×)
Raw	Public	PE	470	250	185,910,044,500	60
	Public	MP	2,000	101	129,905,649,550	42
	This study	MP	5,000	100	83,914,320,400	27
	This study	MP	9,000	100	83,487,871,800	27
	This study	MP	15,000	100	86,438,180,400	28
	Public	10X	400	151	482,628,138,538	156
	Public	PacBio		11,513	238,400,180,650	77
Pre-processed	Public	PE	470	212	125,442,367,208	40
	Public	MP	2,000	86	70,569,596,439	23
	This study	MP	5,000	89	56,873,457,112	18
	This study	MP	9,000	88	55,798,689,316	18
	This study	MP	15,000	87	49,063,748,062	16
	Public	10X	400	140	445,871,690,901	144
	Public	PacBio		11,513	238,400,180,650	77
Platanus-allee input (downsampled)	Public	PE	470	212	125,442,367,208	40
	Public	MP	2,000	86	70,569,596,439	23
	This study	MP	5,000	89	56,873,457,112	18
	This study	MP	9,000	88	55,798,689,316	18
	This study	MP	15,000	87	49,063,748,062	16
	Public	10X	400	140	445,871,690,901	144
	Public	PacBio		11,511	61,990,803,611	20
Synthetic long reads for evaluation	Public	Moleculo		4,026	91,476,572,938	30

Supplementary Table 5. Heterozygosities estimated by GenomeScope based on *k*-mer information.

Species	Estimated heterozygosity (%)
P. polytes (butterfly)	1.52
B. japonicum (amphioxus)	3.48
C. elegans (worm, synthetic	0.38
H. sapiens (human, NA12878)	0.32
A. thaliana (plant, F1 hyblid)	1.05
P. yedoensis (cherry blossom)	2.97
P. alecto (bat)	0.99

For *H. sapiens* (human, NA12878), to obtain the high coverage depth (>100×), the input PE for GenomeScope was the Illumina Platinum library (accession numbers , ERR174330–ERR174339), which was different from the PE for *de novo* assembly in this study.

Supplementary Table 6. NG50 of primary contigs of FALCON-Unzip for various parameters.

Sample	Expected coverage depth	FALCON-integrate-length cutoff, 15000	Chin et al. 2016-length cutoff, 4000	Koren et al. 2018-length cutoff, 4000	Koren et al. 2018-length cutoff, 5000
P. polytes (butterfly)	99	4,403,605	4,686,366	5,065,704	**5,191,218**
B. japonicum (amphioxus)	156	**4,316,335**	1,466,997	1,963,029	1,969,034
C. elegans (worm, synthetic diploid from N2 and	192	**1,702,049**	1,388,672	1,208,219	1,305,369
C. elegans (worm, synthetic diploid from N2 and	160	**1,985,022**	1,421,835	1,770,655	1,714,073
C. elegans (worm, synthetic diploid from N2 and	120	**2,063,029**	1,018,771	1,679,379	1,658,010
C. elegans (worm, synthetic diploid from N2 and	80	**1,185,180**	1,005,714	1,019,936	1,071,206
H. sapiens (human, NA12878)	77	2,049,702	8,474,918	7,879,492	**8,669,871**
C. elegans (worm, fully simulated, heterozygosity	192	**15,247,178**	0	51,276	95,356
C. elegans (worm, fully simulated, heterozygosity	192	**15,235,242**	0	15,919	41,989
C. elegans (worm, fully simulated, heterozygosity	192	**15,212,709**	0	0	10,624
C. elegans (worm, fully simulated, heterozygosity	192	**15,174,627**	0	17,626	34,256

Supplementary Table 7. Phased block statistics for various input data.

Sample	Assembler	Input data	Total (bp)	Scaffold NG50 (bp)	Scaffold LG50 (#)	Contig NG50 (bp)	Contig LG50 (#)	% gaps	BUSCO duplicate complete (%)	% exact-match MP15k pairs
P. polytes (butterfly)	Platanus-allee	PE + PacBio(x20)	463,846,947	369,520	387	92,616	1,482	3.12	87.72	30.95
P. polytes (butterfly)	Platanus-allee	PE + 1 MP(2 kb) + PacBio(x20)	460,610,641	788,623	185	135,163	1,040	1.59	89.15	32.99
B. japonicum (amphioxus)	Platanus-allee	PE + PacBio(x20)	766,087,894	158,419	1,235	20,823	9,267	8.87	82.00	
B. japonicum (amphioxus)	Platanus-allee	PE + 1 MP(3 kb) + PacBio(x20)	728,378,180	646,052	300	38,864	5,225	3.95	87.53	
B. japonicum (amphioxus)	Platanus-allee	PE(x123) + 3 MP(≤10 kb, x105–x123)	734,423,364	945,771	210	62,559	3,297	2.24	86.09	
B. japonicum (amphioxus)	Platanus-allee	PE(x123) + 3 MP(≤10 kb, x105–x123) + PacBio(x156)	758,509,001	1,227,042	187	66,267	3,174	3.02	87.53	
B. japonicum (amphioxus)	Platanus-allee	PE(x123) + 3 MP(≤10 kb, x105–x123) + PacBio(x156) + 10X	769,865,071	1,232,262	191	47,981	4,424	3.11	86.40	
C. elegans (worm, synthetic diploid from N2 and CB4856)	Platanus-allee	PE + PacBio(x20)	203,490,052	104,930	537	41,657	1,353	2.65	89.61	
C. elegans (worm, synthetic diploid from N2 and CB4856)	Platanus-allee	PE + 1 MP(5 kb) + PacBio(x20)	201,307,544	281,192	204	50,696	1,133	2.13	88.70	
C. elegans (worm, synthetic diploid from N2 and CB4856)	FALCON-Unzip	PacBio(x80)	183,701,742	146,065	344	146,065	344	0.00	58.66	
C. elegans (worm, synthetic diploid from N2 and CB4856)	FALCON-Unzip	PacBio(x120)	211,337,801	291,506	174	291,506	174	0.00	74.13	
C. elegans (worm, synthetic diploid from N2 and CB4856)	FALCON-Unzip	PacBio(x160)	230,708,846	416,462	120	416,462	120	0.00	81.57	
C. elegans (worm, synthetic diploid from N2 and CB4856)	FALCON-Unzip, Pilon, PH	PacBio(x80) + PE	180,846,924	145,249	347	145,249	347	0.00	60.18	
C. elegans (worm, synthetic diploid from N2 and CB4856)	FALCON-Unzip, Pilon, PH	PacBio(x120) + PE	207,826,984	294,553	173	294,553	173	0.00	74.54	
C. elegans (worm, synthetic diploid from N2 and CB4856)	FALCON-Unzip, Pilon, PH	PacBio(x160) + PE	222,009,902	416,522	120	416,522	120	0.00	81.98	
H. sapiens (human, NA12878)	Platanus-allee	PE + PacBio(x20)	5,624,020,712	560,522	2,635	24,549	68,533	2.36	72.71	
H. sapiens (human, NA12878)	Platanus-allee	PE + 1 MP(2 kb) + PacBio(x20)	5,584,927,756	750,838	2,052	24,706	66,868	1.85	70.96	

Supplementary Table 8. Consensus sequence statistics for various input data.

Sample	Assembler	Input data	Total (bp)	Scaffold NG50 (bp)	Scaffold LG50 (#)	Contig NG50 (bp)	Contig LG50 (#)	% gaps	BUSCO single complete (%)
P. polytes (butterfly)	Platanus-allee	PE + PacBio(x20)	254,608,813	609,090	127	98,302	722	4.23	96.19
		PE + 1 MP(2 kb) + [illegible]	245,819,409	1,364,255	54	138,842	508	2.08	96.56
B. japonicum (amphioxus)	Platanus-allee	PE + PacBio(x20)	437,941,749	278,100	355	22,011	4573	11.89	93.97
		PE + 1 MP(3 kb) +	388,129,624	1,644,498	57	39,548	2616	4.96	94.99
		PE(x123) + 3 MP(≤10 kb, x105–x123)	396,319,232	5,118,024	25	66,558	1586	2.78	95.09
		PE(x123) + 3 MP(≤10 kb, x105–x123) + PacBio(x156)	403,693,174	7,007,161	19	67,783	1581	3.96	94.17
		PE(x123) + 3 MP(≤10 kb, x105–x123) + PacBio(x156) + [illegible]	411,842,385	5,024,185	21	49,606	2191	3.94	93.97
C. elegans (worm, synthetic diploid from N2 and CB4856)	Platanus-allee	PE + 3 MP(≤16 kb)	105,819,652	2,387,679	14	62,889	458	4.76	95.52
		PE + PacBio(x20)	109,069,094	539,738	51	60,670	469	3.39	96.44
		PE + 1 MP(5 kb) +	104,994,001	1,860,654	17	60,250	470	2.22	96.03
		PE + 3 MP(≤16 kb) +	106,543,206	3,315,597	12	64,210	456	5.13	95.11
	Platanus (v1.2.4)	PE + 3 MP(≤16 kb)	101,639,826	1,848,059	19	71,193	364	2.30	96.64
	FALCON-Unzip	PacBio(x80)	100,914,967	1,188,014	23	1,188,014	23	0.00	89.21
		PacBio(x120)	106,422,493	1,715,058	20	1,715,058	20	0.00	93.08
		PacBio(x160)	109,274,681	1,968,624	19	1,968,624	19	0.00	93.18
	FALCON-Unzip, Pilon, PH	PacBio(x80) + PE	97,867,472	1,188,225	23	1,188,225	23	0.00	90.63
		PacBio(x120) + PE	101,732,861	1,714,203	20	1,714,203	20	0.00	95.11
		PacBio(x160) + PE	103,134,152	1,968,772	19	1,968,772	19	0.00	95.01
H. sapiens (human,	Platanus-allee	PE + PacBio(x20)	2,895,343,257	1,293,823	711	26,230	32,510	2.60	90.50
		PE + 1 MP(2 kb) +	2,877,319,935	2,633,140	328	26,299	31,878	1.85	90.15

Supplementary Table 9. Real run time of Platanus-allee.

Sample	Input data	Time for each command (hour)			Total (hour)
		assemble (input, PE only)	phase	consensus	
P. polytes (butterfly)	PE + 4 MP(≤15 kb)	0.99	13.49	0.99	15.48
	PE + PacBio(x20)	0.55	7.88	0.55	8.98
	PE + 1 MP(2 kb) + PacBio(x20)	0.65	12.85	0.65	14.14
	PE + 4 MP(≤15 kb) + PacBio(x20)	1.15	18.29	1.15	20.58
	PE + 4 MP(≤15 kb) + 10X	2.67	24.34	2.67	29.68
	PE + 4 MP(≤15 kb) + PacBio(x20) +	3.92	32.82	3.92	40.66
B. japonicum (amphioxus)	PE + 3 MP(≤10 kb)	3.32	30.76	0.42	34.51
	PE + PacBio(x20)	3.32	60.29	1.45	65.06
	PE + 1 MP(3 kb) + PacBio(x20)	3.32	50.88	1.31	55.51
	PE + 3 MP(≤10 kb) + PacBio(x20)	3.32	64.82	1.43	69.57
	PE + 3 MP(≤10 kb) + 10X	3.32	40.97	0.79	45.09
	PE + 3 MP(≤10 kb) + PacBio(x20) + 10X	3.32	81.99	1.79	87.10
C. elegans (worm, synthetic diploid from N2 and CB4856)	PE + 3 MP(≤16 kb)	2.36	4.07	0.12	6.55
	PE + PacBio(x20)	2.36	4.00	0.08	6.44
	PE + 1 MP(5 kb) + PacBio(x20)	2.36	4.31	0.10	6.78
	PE + 3 MP(≤16 kb) + PacBio(x20)	2.36	5.96	0.13	8.45
H. sapiens (human, NA12878)	PE + 4 MP(≤15 kb)	28.01	154.88	7.12	190.01
	PE + PacBio(x20)	28.01	76.97	4.02	109.00
	PE + 1 MP(2 kb) + PacBio(x20)	28.01	102.86	5.24	136.11
	PE + 4 MP(≤15 kb) + PacBio(x20)	28.01	204.44	7.18	239.63
	PE + 4 MP(≤15 kb) + 10X	28.01	210.36	20.03	258.40
	PE + 4 MP(≤15 kb) + PacBio(x20) +	28.01	277.18	19.39	324.58

Supplementary Table 10. CPU run time of Platanus-allee.

Sample	Input data	Time for each command (hour)			Total (hour)
		assemble (input, PE only)	phase	consensus	
P. polytes (butterfly)	PE + 4 MP(≤15 kb)	14.92	166.15	13.54	194.60
	PE + PacBio(x20)	14.92	63.98	6.56	85.46
	PE + 1 MP(2 kb) + PacBio(x20)	14.92	103.22	8.68	126.81
	PE + 4 MP(≤15 kb) + PacBio(x20)	14.92	222.40	16.13	253.45
	PE + 4 MP(≤15 kb) + 10X	14.92	216.00	35.38	266.30
	PE + 4 MP(≤15 kb) + PacBio(x20) +	14.92	297.14	50.32	362.37
B. japonicum (amphioxus)	PE + 3 MP(≤10 kb)	13.53	268.98	2.50	285.01
	PE + PacBio(x20)	13.53	509.70	20.43	543.66
	PE + 1 MP(3 kb) + PacBio(x20)	13.53	441.51	17.51	472.55
	PE + 3 MP(≤10 kb) + PacBio(x20)	13.53	615.81	19.06	648.40
	PE + 3 MP(≤10 kb) + 10X	13.53	338.84	5.95	358.33
	PE + 3 MP(≤10 kb) + PacBio(x20) + 10X	13.53	750.74	22.25	786.52
C. elegans (worm, synthetic diploid from N2 and CB4856)	PE + 3 MP(≤16 kb)	5.67	40.50	0.86	47.03
	PE + PacBio(x20)	5.67	32.47	0.57	38.71
	PE + 1 MP(5 kb) + PacBio(x20)	5.67	35.57	0.75	41.99
	PE + 3 MP(≤16 kb) + PacBio(x20)	5.67	61.23	1.22	68.13
H. sapiens (human, NA12878)	PE + 4 MP(≤15 kb)	90.40	1804.96	88.83	1984.18
	PE + PacBio(x20)	90.40	644.04	32.76	767.19
	PE + 1 MP(2 kb) + PacBio(x20)	90.40	986.58	50.44	1127.41
	PE + 4 MP(≤15 kb) + PacBio(x20)	90.40	2486.08	92.17	2668.64
	PE + 4 MP(≤15 kb) + 10X	90.40	2201.47	247.68	2539.54
	PE + 4 MP(≤15 kb) + PacBio(x20) +	90.40	3079.17	240.79	3410.36

Supplementary Table 11. Evaluation of *B. japonicum* phased blocks based on synthetic long reads.

Evaluation fragment size (bp)	Assembler	Input data	Recall (%)	Precision (%)	F-measure (%)
1k	Platanus-allee	PE + 3 MP(≤10 kb)	72.309	88.315	79.515
		PE + PacBio(x20)	67.527	87.665	76.290
		PE + 1 MP(3 kb) + PacBio(x20)	71.556	88.245	79.029
		PE + 3 MP(≤10 kb) + PacBio(x20)	72.580	88.035	79.564
		PE + 3 MP(≤10 kb) + 10X	71.021	87.710	78.488
		PE + 3 MP(≤10 kb) + PacBio(x20) + 10X	71.249	87.444	78.520
		PE(x123) + 3 MP(≤10 kb, x105–x123)	75.358	**88.393**	**81.357**
		PE(x123) + 3 MP(≤10 kb, x105–x123) + PacBio(x156)	**75.536**	88.074	81.324
		PE(x123) + 3 MP(≤10 kb, x105–x123) + PacBio(x156) + 10X	74.749	87.510	80.628
	FALCON-Unzip	PacBio(x156)	68.721	66.609	67.649
	FALCON-Unzip, Pilon, PH	PacBio(x156) + PE	74.900	72.356	73.606
	Supernova	10X	52.761	84.087	64.838
5k	Platanus-allee	PE + 3 MP(≤10 kb)	37.979	47.391	42.166
		PE + PacBio(x20)	31.844	48.830	38.549
		PE + 1 MP(3 kb) + PacBio(x20)	37.218	47.988	41.923
		PE + 3 MP(≤10 kb) + PacBio(x20)	38.226	47.037	42.176
		PE + 3 MP(≤10 kb) + 10X	35.449	46.792	40.339
		PE + 3 MP(≤10 kb) + PacBio(x20) + 10X	35.639	46.601	40.389
		PE(x123) + 3 MP(≤10 kb, x105–x123)	41.498	**48.258**	**44.624**
		PE(x123) + 3 MP(≤10 kb, x105–x123) + PacBio(x156)	**41.753**	47.616	44.492
		PE(x123) + 3 MP(≤10 kb, x105–x123) + PacBio(x156) + 10X	40.010	47.212	43.313
	FALCON-Unzip	PacBio(x156)	31.572	26.350	28.725
	FALCON-Unzip, Pilon, PH	PacBio(x156) + PE	38.910	31.696	34.934
	Supernova	10X	19.329	39.973	26.058

Supplementary Table 12. Evaluation of phased blocks of the synthetic diploid sample (*C. elegans*) based on the reference genomes.

Evaluation fragment size (bp)	Assembler	Input data	Recall (%)	Precision (%)	F-measure (%)
1k	Platanus-allee	PE + 3 MP(≤16 kb)	84.420	92.371	88.217
		PE + PacBio(x20)	85.122	91.928	88.394
		PE + 1 MP(5 kb) + PacBio(x20)	**86.759**	92.277	89.433
		PE + 3 MP(≤16 kb) + PacBio(x20)	86.688	**92.375**	**89.441**
	FALCON-Unzip	PacBio(x80)	63.674	62.778	63.223
		PacBio(x120)	74.721	68.987	71.739
		PacBio(x160)	78.950	70.193	74.315
		PacBio(x192)	80.603	70.708	75.332
	FALCON-Unzip, Pilon, PH	PacBio(x80) + PE	74.026	74.346	74.186
		PacBio(x120) + PE	82.157	76.761	79.367
		PacBio(x160) + PE	84.756	76.399	80.361
		PacBio(x192) + PE	85.747	76.282	80.738
5k	Platanus-allee	PE + 3 MP(≤16 kb)	56.809	**76.888**	65.341
		PE + PacBio(x20)	46.221	59.435	52.002
		PE + 1 MP(5 kb) + PacBio(x20)	57.940	72.178	64.280
		PE + 3 MP(≤16 kb) + PacBio(x20)	**62.032**	76.262	**68.415**
	FALCON-Unzip	PacBio(x80)	26.405	28.791	27.546
		PacBio(x120)	40.418	39.499	39.953
		PacBio(x160)	47.934	43.871	45.812
		PacBio(x192)	50.837	45.384	47.956
	FALCON-Unzip, Pilon, PH	PacBio(x80) + PE	39.811	44.476	42.014
		PacBio(x120) + PE	51.512	51.173	51.342
		PacBio(x160) + PE	57.116	53.042	55.004
		PacBio(x192) + PE	59.081	53.722	56.274

Supplementary Table 13. Evaluation of the errors in phased blocks for *C. elegans* synthetic diploid data.

Evaluation fragment size (bp)	Assembler	Input data	Aligned fragments (bp)	% identity	# switch-errors	# mis-assemblies	# switch-error rate /fragment	# mis-assemblies /fragment	# (switch + mis-assemblies) /fragment
1k	Platanus-allee	PE + 3 MP(≤16 kb)	175,430,000	99.934	4,282	1,093	**0.02441**	0.00623	**0.03064**
		PE + PacBio(x20)	188,113,000	**99.935**	20,042	805	0.10654	**0.00428**	0.11082
		PE + 1 MP(5 kb) +	188,379,000	99.933	10,081	1,093	0.05351	0.00580	0.05932
		PE + 3 MP(≤16 kb) +	185,805,000	99.932	5,903	1,218	0.03177	0.00656	0.03833
	FALCON-Unzip	PacBio(x80)	179,819,000	99.795	5,752	1,859	0.03199	0.01034	0.04233
		PacBio(x120)	206,807,000	99.808	6,245	2,381	0.03020	0.01151	0.04171
		PacBio(x160)	225,408,000	99.795	6,748	2,903	0.02994	0.01288	0.04282
		PacBio(x192)	236,971,000	99.792	6,955	3,051	0.02935	0.01287	0.04222
	FALCON-Unzip, Pilon, PH	PacBio(x80) + PE	177,091,000	99.825	6,434	1,869	0.03633	0.01055	0.04689
		PacBio(x120) + PE	203,476,000	99.830	6,354	2,351	0.03123	0.01155	0.04278
		PacBio(x160) + PE	217,030,000	99.816	6,620	2,893	0.03050	0.01333	0.04383
		PacBio(x192) + PE	226,887,000	99.813	6,686	2,977	0.02947	0.01312	0.04259
10k	Platanus-allee	PE + 3 MP(≤16 kb)	143,480,000	99.962	455	13	**0.03171**	**0.00091**	**0.03262**
		PE + PacBio(x20)	159,400,000	99.959	2,417	40	0.15163	0.00251	0.15414
		PE + 1 MP(5 kb) +	167,210,000	99.958	1,907	21	0.11405	0.00126	0.11530
		PE + 3 MP(≤16 kb) +	160,840,000	**99.962**	933	28	0.05801	0.00174	0.05975
	FALCON-Unzip	PacBio(x80)	163,910,000	99.871	781	40	0.04765	0.00244	0.05009
		PacBio(x120)	189,640,000	99.882	858	50	0.04524	0.00264	0.04788
		PacBio(x160)	205,400,000	99.879	964	45	0.04693	0.00219	0.04912
		PacBio(x192)	215,580,000	99.874	965	48	0.04476	0.00223	0.04699
	FALCON-Unzip, Pilon, PH	PacBio(x80) + PE	161,850,000	99.896	821	37	0.05073	0.00229	0.05301
		PacBio(x120) + PE	187,430,000	99.901	859	44	0.04583	0.00235	0.04818
		PacBio(x160) + PE	199,520,000	99.892	962	50	0.04822	0.00251	0.05072
		PacBio(x192) + PE	208,590,000	99.889	965	54	0.04626	0.00259	0.04885

Supplementary Table 14. Accuracy evaluation of consensus scaffolds for *C. elegans* synthetic diploid data.

Evaluation fragment size (bp)	Assembler	Input data	Aligned fragments (bp)	% identity	# mis-assemblies	# mis-assemblies /fragment
1k	Platanus-allee	PE + 3 MP(≤16 kb)	94,661,000	99.766	1,129	0.01193
		PE + PacBio(x20)	98,873,000	99.756	1,007	0.01018
		PE + 1 MP(5 kb) + PacBio(x20)	96,709,000	99.764	1,119	0.01157
		PE + 3 MP(≤16 kb) +	94,454,000	99.731	1,271	0.01346
	Platanus (v1.2.4)	PE + 3 MP(≤16 kb)	94,528,000	**99.790**	**738**	**0.00781**
	FALCON-Unzip	PacBio(x80)	98,468,000	99.725	1,555	0.01579
		PacBio(x120)	103,831,000	99.738	1,751	0.01686
		PacBio(x160)	106,321,000	99.699	2,105	0.01980
		PacBio(x192)	106,265,000	99.693	2,276	0.02142
	FALCON-Unzip, Pilon, PH	PacBio(x80) + PE	95,872,000	99.781	1,358	0.01416
		PacBio(x120) + PE	99,491,000	99.777	1,570	0.01578
		PacBio(x160) + PE	100,658,000	99.738	1,781	0.01769
		PacBio(x192) + PE	100,568,000	99.743	1,986	0.01975
10k	Platanus-allee	PE + 3 MP(≤16 kb)	81,520,000	99.835	**18**	**0.00221**
		PE + PacBio(x20)	88,540,000	99.833	41	0.00463
		PE + 1 MP(5 kb) + PacBio(x20)	87,660,000	99.822	32	0.00365
		PE + 3 MP(≤16 kb) +	81,230,000	99.794	28	0.00345
	Platanus (v1.2.4)	PE + 3 MP(≤16 kb)	88,100,000	99.854	26	0.00295
	FALCON-Unzip	PacBio(x80)	93,960,000	99.825	39	0.00415
		PacBio(x120)	99,160,000	99.833	55	0.00555
		PacBio(x160)	101,120,000	99.808	47	0.00465
		PacBio(x192)	100,860,000	99.807	53	0.00525
	FALCON-Unzip, Pilon, PH	PacBio(x80) + PE	91,990,000	99.869	34	0.00370
		PacBio(x120) + PE	95,440,000	**99.870**	47	0.00492
		PacBio(x160) + PE	96,360,000	99.837	43	0.00446
		PacBio(x192) + PE	96,060,000	99.842	51	0.00531

Supplementary Table 15. Evaluation of *H. sapiens* phased blocks based on synthetic long reads.

Evaluation fragment size (bp)	Assembler	Input data	Recall (%)	Precision (%)	F-measure (%)
1k	Platanus-allee	PE + 4 MP(≤15 kb)	59.204	91.781	71.978
		PE + PacBio(x20)	73.097	90.642	80.930
		PE + 1 MP(2 kb) + PacBio(x20)	72.758	90.925	80.833
		PE + 4 MP(≤15 kb) + PacBio(x20)	69.313	91.196	78.763
		PE + 4 MP(≤15 kb) + 10X	71.328	**92.204**	80.433
		PE + 4 MP(≤15 kb) + PacBio(x20) +	73.245	91.934	**81.532**
	FALCON-Unzip	PacBio(x77)	59.885	60.195	60.039
	FALCON-Unzip, Pilon,	PacBio(x77) + PE	66.694	68.751	67.707
	Supernova	10X	**75.456**	88.590	81.497
	Mostovoy et al. 2016	PE + 1 MP(2 kb)+ 10X + Bionano	61.763	85.425	71.692
5k	Platanus-allee	PE + 4 MP(≤15 kb)	14.702	49.867	22.709
		PE + PacBio(x20)	23.790	37.685	29.167
		PE + 1 MP(2 kb) + PacBio(x20)	25.868	41.249	31.796
		PE + 4 MP(≤15 kb) + PacBio(x20)	24.185	43.333	31.044
		PE + 4 MP(≤15 kb) + 10X	27.199	**50.710**	35.407
		PE + 4 MP(≤15 kb) + PacBio(x20) +	30.139	48.950	**37.308**
	FALCON-Unzip	PacBio(x77)	14.652	15.494	15.061
	FALCON-Unzip, Pilon,	PacBio(x77) + PE	19.406	21.527	20.412
	Supernova	10X	**32.956**	39.447	35.911
	Mostovoy et al. 2016	PE + 1 MP(2 kb)+ 10X + Bionano	17.819	42.509	25.111

Supplementary Table 16. Evaluation of human NA12878 phased blocks based on the Platinum data (Eberle *et al.*, 2017[16]).

Evaluation fragment size (bp)	Assembler	Input data	Recall (%)	Precision (%)	F-measure (%)
1k	Platanus-allee	PE + 3 MP(≤10 kb)	63.382	90.009	74.384
		PE + PacBio(x20)	78.503	89.124	83.477
		PE + 1 MP(3 kb) + PacBio(x20)	78.090	89.362	83.346
		PE + 3 MP(≤10 kb) + PacBio(x20)	74.297	89.575	81.224
		PE + 3 MP(≤10 kb) + 10X	76.034	**90.605**	82.682
		PE + 3 MP(≤10 kb) + PacBio(x20) + 10X	78.258	90.298	83.848
	FALCON-Unzip	PacBio(x77)	63.852	58.217	60.905
	FALCON-Unzip, Pilon, PH	PacBio(x77) + PE	72.398	66.845	69.511
	Supernova	10X	**82.753**	86.971	**84.810**
	Mostovoy et al. 2016	PE + 1 MP(2 kb)+ 10X + Bionano	62.598	83.634	71.603
5k	Platanus-allee	PE + 3 MP(≤10 kb)	20.913	62.251	31.308
		PE + PacBio(x20)	34.022	49.055	40.178
		PE + 1 MP(3 kb) + PacBio(x20)	36.722	53.714	43.622
		PE + 3 MP(≤10 kb) + PacBio(x20)	34.590	56.485	42.905
		PE + 3 MP(≤10 kb) + 10X	37.464	**65.516**	47.670
		PE + 3 MP(≤10 kb) + PacBio(x20) + 10X	42.006	63.474	50.556
	FALCON-Unzip	PacBio(x77)	20.771	20.079	20.419
	FALCON-Unzip, Pilon, PH	PacBio(x77) + PE	28.692	28.755	28.723
	Supernova	10X	**49.421**	54.346	**51.767**
	Mostovoy et al. 2016	PE + 1 MP(2 kb)+ 10X + Bionano	21.317	49.853	29.864

Supplementary Table 17. Evaluation of the errors in phased blocks for human NA12878 based on the Platinum data (Eberle *et al.*, 2017[16]).

Evaluation fragment size (bp)	Assembler	Input data	Aligned fragments (bp)	% identity	# switch-errors	# mis-assemblies	# switch-errors /fragment	# mis-assemblies /fragment	# (switch + mis-assemblies)
1k	Platanus-allee	PE + 4 MP(≤15 kb)	3,144,055,000	99.896	120,656	8,032	0.03838	0.00255	0.04093
		PE + PacBio(x20)	5,355,073,000	99.909	638,001	11,125	0.11914	0.00208	0.12122
		PE + 1 MP(2 kb) + PacBio(x20)	5,317,781,000	99.905	509,426	18,135	0.09580	0.00341	0.09921
		PE + 4 MP(≤15 kb) + PacBio(x20)	5,017,992,000	99.902	423,405	18,519	0.08438	0.00369	0.08807
		PE + 4 MP(≤15 kb) + 10X	4,561,230,000	99.905	138,658	13,893	0.03040	0.00305	0.03345
		PE + 4 MP(≤15 kb) + PacBio(x20)	5,281,326,000	99.903	249,916	19,186	0.04732	0.00363	0.05095
	FALCON-Unzip	PacBio(x77)	4,680,464,000	99.783	96,337	12,918	**0.02058**	0.00276	**0.02334**
	FALCON-Unzip, Pilon, PH	PacBio(x77) + PE	4,813,777,000	99.814	103,704	12,469	0.02154	0.00259	0.02413
	Supernova	10X	5,294,378,000	99.914	115,869	10,844	0.02189	0.00205	0.02393
	Mostovoy et al. 2016	PE + 1 MP(2 kb)+ 10X + Bionano	4,638,039,000	**99.935**	157,922	6,867	0.03405	**0.00148**	0.03553
5k	Platanus-allee	PE + 4 MP(≤15 kb)	1,482,700,000	99.933	5,031	123	0.03393	0.00083	0.03476
		PE + PacBio(x20)	4,969,480,000	99.953	124,361	2,459	0.25025	0.00495	0.25520
		PE + 1 MP(2 kb) + PacBio(x20)	4,992,090,000	99.946	119,323	2,985	0.23902	0.00598	0.24500
		PE + 4 MP(≤15 kb) + PacBio(x20)	4,264,470,000	99.947	97,511	2,434	0.22866	0.00571	0.23437
		PE + 4 MP(≤15 kb) + 10X	3,720,360,000	99.954	13,067	448	0.03512	0.00120	0.03633
		PE + 4 MP(≤15 kb) + PacBio(x20)	4,776,820,000	99.953	74,150	2,582	0.15523	0.00541	0.16063
	FALCON-Unzip	PacBio(x77)	4,435,170,000	99.843	28,276	1,053	0.06375	0.00237	0.06613
	FALCON-Unzip, Pilon, PH	PacBio(x77) + PE	4,584,210,000	99.868	30,512	1,060	0.06656	0.00231	0.06887
	Supernova	10X	5,156,060,000	**99.971**	12,364	741	**0.02398**	0.00144	**0.02542**
	Mostovoy et al. 2016	PE + 1 MP(2 kb)+ 10X + Bionano	4,617,680,000	99.969	21,731	320	0.04706	**0.00069**	0.04775

Supplementary Table 18. Evaluation of MHC (HLA) loci of human NA12878 based on the previous typing results (Dilthey *et al*., 2016[17]).

Assembler	Input data	CDS-based (6 loci)		mRNA-based (6 loci)	
		# correct typings	# exact-matches in correct typings	# correct typings	# exact-matches in correct typings
Platanus-allee	PE + 4 MP(≤15 kb)	**6**	**12**	**6**	**8**
	PE + PacBio(x20)	5	10	5	5
	PE + 1 MP(2 kb) + PacBio(x20)	**6**	**12**	**6**	7
	PE + 4 MP(≤15 kb) + PacBio(x20)	**6**	**12**	**6**	**8**
	PE + 4 MP(≤15 kb) + 10X	5	10	5	6
	PE + 4 MP(≤15 kb) + PacBio(x20) + 10X	5	10	5	6
FALCON-Unzip	PacBio(x77)	**6**	8	**6**	1
FALCON-Unzip, Pilon, PH	PacBio(x77) + PE	**6**	11	**6**	5
Supernova	10X	3	6	2	2
Mostovoy et al. 2016	PE + 1 MP(2 kb)+ 10X + Bionano	0	0	0	0

Supplementary Table 19. Platanus-allee bubbles not found in the reference and the other assemblies for human data (NA12878).

Bubble ID	Total length (bp)	% gaps	% unaligned-1k-mer				
			Reference (GRCh38)	FALCON-Unzip	FALCON-Unzip, Pilon, PH	Supernova	Mostovoy et al. 2016
bubble33180	816,455	16.77	65.31	52.72	54.93	27.04	79.76
bubble43779	458,260	14.00	47.02	60.71	61.31	28.57	81.25
bubble50544	434,885	14.20	71.92	47.00	51.74	34.39	79.81
bubble56428	304,310	12.40	78.99	78.99	78.99	27.73	78.99
bubble48643	264,534	26.64	73.88	73.88	73.88	41.05	73.88
bubble45565	137,327	19.80	80.00	80.00	80.00	34.12	80.00
bubble64527	117,616	20.85	62.50	62.50	62.50	30.56	62.50
bubble49433	109,173	23.84	61.11	61.11	61.11	31.48	61.11

Supplementary Table 20. Sequencing reads of *A. thaliana F1-hybrid* from public databases.

Status	Library type	Nominal insert length (bp)	Mean read length (bp)	Total length (bp)	Expected sequence coverage (×)
Raw	PE	400	249	8,105,209,901	60
	PacBio		17,525	11,084,590,243	82
Pre-processed	PE	400	233	7,518,292,592	55
	PacBio		17,525	11,084,590,243	82
Platanus-allee input (downsampled)	PE	400	233	7,518,292,592	55
	PacBio		17,550	2,722,817,447	20

Supplementary Table 21. Sequencing reads of *P. yedonensis* (cherry blossom) from public databases.

Description	Library type	Nominal insert length (bp)	Mean read length (bp)	Total length (bp)	Expected sequence coverage (×)
Raw	PE	250	151	38,096,485,808	141
	PE	500	300	33,682,954,486	125
	MP	3,000	149	21,550,093,231	80
	MP	5,000	148	23,660,690,862	88
	MP	10,000	148	21,292,872,596	79
	MP	15,000	150	23,541,425,847	87
	PacBio		9,575	18,769,924,156	70
Pre-processed	PE	250	137	31,940,003,998	118
	PE	500	269	29,324,326,879	109
	MP	3,000	92	7,067,814,658	26
	MP	5,000	93	7,785,428,855	29
	MP	10,000	95	7,321,558,722	27
	MP	15,000	103	9,926,543,451	37
	PacBio		9,575	18,769,924,156	70
Platanus-allee input (downsampled)	PE	250	137	10,799,731,851	40
	PE	500	269	10,799,662,966	40
	MP	3,000	92	7,067,814,658	26
	MP	5,000	93	7,785,428,855	29
	MP	10,000	95	7,321,558,722	27
	MP	15,000	103	9,926,543,451	37
	PacBio		9,578	5,401,667,766	20

Supplementary Table 22. Sequencing reads of *P. alecto* (bat) from public databases.

Description	Library type	Nominal insert length (bp)	Mean read length (bp)	Total length (bp)	Expected sequence coverage (×)
Raw	PE	250	150	95,280,395,100	45
	PE	500	150	54,370,638,000	26
	PE	800	150	46,324,101,000	22
	MP	2,000	49	35,360,549,434	17
	MP	5,000	49	35,154,428,876	17
	MP	10,000	49	33,680,270,638	16
	MP	20,000	49	35,964,061,168	17
Pre-processed	PE	250	144	89,231,100,006	42
	PE	500	139	48,425,702,281	23
	PE	800	112	31,881,421,136	15
	MP	2,000	47	31,821,770,893	15
	MP	5,000	47	31,648,919,140	15
	MP	10,000	48	30,548,047,773	15
	MP	20,000	47	32,104,866,704	15

Supplementary Table 23. Phased block statistics for benchmarks using public data.

Sample	Assembler	Input data	Total (bp)	Scaffold NG50 (bp)	Scaffold LG50 (#)	Contig NG50 (bp)	Contig LG50 (#)	% gaps	BUSCO duplicate complete (%)
Arabidopsis thaliana (F1 hyblid)	Platanus-allee	PE + PacBio(x20)	261,503,785	77,360	967	26,107	2,710	7.06	83.33
	Public (FALCON-Unzip)	PacBio(x82)	247,188,744	**2,962,956**	**19**	**2,962,956**	**19**	**0.00**	**93.33**
Prunus yedoensis (cherry blossum)	Platanus-allee	PE + 4 MP(≤15 kb)	493,191,504	293,134	377	21,584	6,091	3.63	89.24
		PE + PacBio(x20)	533,104,610	50,072	2,442	17,202	7,647	5.51	88.13
		PE + 1 MP(3 kb) + PacBio(x20)	520,213,742	150,285	840	22,416	6,110	**2.54**	**89.38**
		PE + 4 MP(≤15 kb) + PacBio(x20)	547,167,377	**775,582**	**171**	**25,098**	**5,671**	4.06	89.10
	Public (FALCON-based, consensus)	PE + 5 MP(≤20 kb) + PacBio(x70) + Fosmid-ends	319,209,792	178,190	481	**157,043**	**543**	**0.15**	28.19
Pteropus alecto (bat)	Platanus-allee	PE + 4 MP(≤20 kb)	3,746,739,676	**342,236**	**2,698**	19,185	57,731	5.52	**56.28**
H. sapiens (human, NA12878)	Public (FALCON-Unzip)	PacBio(x77)	4,721,106,192	108,668	13,508	**108,668**	**13,508**	**0.00**	30.36

Statistics were calculated for phased blocks with length ≥500 bp. NG50s and LG50s were calculated based on the estimated diploid genome sizes (*A. thaliana*, 260 Mbp; *P. yedonensis*, 540 Mbp; *P. alecto*, 4.2 Gbp; *H. sapiens* 6.2 Gbp). BUSCO version 3.0.2 was used to estimate the rate of the phased single-copy genes. For *A. thaliana, P. yedonensis,* embryophyta set (1440 orthologs) was used. For *P. alecto* and *H. sapiens,* the euarchotoglires set (6192 orthologs) was used.

Supplementary Table 24. Consensus sequence statistics for benchmarks using public data.

Sample	Assembler	Input data	Total (bp)	Scaffold NG50 (bp)	Scaffold LG50 (#)	Contig NG50 (bp)	Contig LG50 (#)	% gaps	BUSCO single complete (%)
Arabidopsis thaliana (F1 hyblid)	Platanus-allee	PE + PacBio(x20)	149,452,430	292,434	127	36,827	1,037	8.97	94.03
	Platanus (v1.2.4)	PE	122,322,782	29,081	1,111	22,255	1,562	0.05	**96.74**
	Public (FALCON-Unzip)	PacBio(x82)	140,024,976	**7,960,654**	**7**	**7,960,654**	**7**	**0.00**	91.94
Prunus yedoensis (cherry blossum)	Platanus-allee	PE + 4 MP(≤15 kb)	309,342,597	**2,123,503**	**38**	26,772	2,754	4.15	94.10
		PE + PacBio(x20)	356,772,672	106,090	715	24,095	3,186	8.07	93.82
		PE + 1 MP(3 kb) +	319,250,950	281,046	277	26,983	2,776	3.29	**94.44**
		PE + 4 MP(≤15 kb) +	309,344,527	1,727,581	43	27,187	2,723	4.29	93.89
	Platanus (v1.2.4)	PE + 4 MP(≤15 kb)	350,766,246	431,649	181	10,491	7,518	11.78	82.36
	Public (FALCON-based)	PE + 5 MP(≤20 kb) + PacBio(x70) + Fosmid-ends	319,209,792	178,190	481	**157,043**	**543**	**0.15**	59.38
Pteropus alecto (bat)	Platanus-allee	PE + 4 MP(≤20 kb)	2,167,287,614	18,284,809	34	25,932	22,986	5.23	87.47
	Platanus (v1.2.4)	PE + 4 MP(≤20 kb)	1,989,876,835	**24,402,672**	**28**	**37,716**	**16,123**	**1.52**	**92.36**
	Public (SOAPdenovo-based)	PE + 4 MP(≤20 kb)	1,985,975,446	14,933,599	39	29,198	20,866	2.08	91.89
H. sapiens (human, NA12878)	Public (FALCON-Unzip)	PacBio(x77)	2,787,672,821	8,669,871	98	8,669,871	98	0.00	87.97

Statistics were calculated for consensus sequences with length ≥500 bp. NG50s and LG50s were calculated based on the estimated diploid genome sizes (*A. thaliana*, 130 Mbp; *P. yedonensis*, 270 Mbp; *P. alecto*, 2.1 Gbp; *H. sapiens* 3.1 Gbp). BUSCO version 3.0.2 was used to estimate the rate of the single-copy genes. For *A. thaliana, P. yedonensis,* embryophyta set (1440 orthologs) was used. For *P. alecto* and *H. sapiens,* the euarchotoglires set (6192 orthologs) was used.

Supplementary Table 25. Evaluation of *A. thaliana* (F1 hybrid) phased blocks.

Evaluation fragment size (bp)	Assembler	Input data	Recall (%)	Precision (%)	F-measure (%)
1k	Platanus-allee	PE + PacBio(x20)	80.397	**91.427**	85.558
	FALCON-Unzip	PacBio(x82)	**89.289**	87.702	**88.488**
5k	Platanus-allee	PE + PacBio(x20)	44.344	63.804	52.323
	FALCON-Unzip	PacBio(x82)	**66.231**	**64.991**	**65.605**

Supplementary Table 26. Phased block statistics for fully simulated data of *C. elegans*.

Simulated heterozygosity (%)	Assembler	Input data	Total (bp)	Scaffold NG50 (bp)	Scaffold LG50 (#)	Contig NG50 (bp)	Contig LG50 (#)	% gaps	BUSCO duplicate complete (%)
0.1	Platanus-allee	PE + 4 MP(≤15 kb)	204,913,212	579,095	105	94,792	573	7.76	13.34
	FALCON-Unzip	PacBio(x192)	203,686,079	10,803,006	8	**10,803,006**	**8**	**0.00**	9.57
	FALCON-Unzip, Pilon, PH	PacBio(x192) + PE	182,346,398	10,115,753	8	10,115,753	**8**	**0.00**	**20.98**
	Supernova	10X	200,090,889	**13,444,511**	**6**	150,497	354	1.68	7.64
0.5	Platanus-allee	PE + 4 MP(≤15 kb)	202,412,579	8,618,403	9	343,714	173	0.96	32.49
	FALCON-Unzip	PacBio(x192)	176,944,385	11,431,407	**7**	11,431,407	**7**	**0.00**	27.29
	FALCON-Unzip, Pilon, PH	PacBio(x192) + PE	197,300,668	**13,275,475**	**7**	**13,275,475**	**7**	**0.00**	**34.62**
	Supernova	10X	201,199,160	12,328,346	**7**	143,918	369	1.79	32.08
1.0	Platanus-allee	PE + 4 MP(≤15 kb)	200,921,232	**17,420,676**	**6**	515,871	112	0.44	52.65
	FALCON-Unzip	PacBio(x192)	205,411,733	13,205,881	7	**13,205,881**	**7**	**0.00**	53.26
	FALCON-Unzip, Pilon, PH	PacBio(x192) + PE	198,460,414	13,205,881	7	**13,205,881**	**7**	**0.00**	**54.99**
	Supernova	10X	207,782,148	6,277,788	10	127,932	411	2.59	52.14
2.0	Platanus-allee	PE + 4 MP(≤15 kb)	201,006,438	**15,573,342**	**6**	731,834	66	0.34	**83.50**
	FALCON-Unzip	PacBio(x192)	204,564,658	14,731,613	7	**14,731,613**	**7**	**0.00**	82.08
	FALCON-Unzip, Pilon, PH	PacBio(x192) + PE	197,258,927	14,731,613	7	**14,731,613**	**7**	**0.00**	81.16
	Supernova	10X	174,296,828	45,771	709	32,315	1,311	3.05	71.08

The cause of the small BUSCO values is not clear. **Supplementary Table 27** is more informative for the completeness.

Supplementary Table 27. Evaluation of phased blocks for fully simulated data of *C. elegans* based on the reference genome.

Evaluation fragment size (bp)	Simulated heterozygosity (%)	Assembler	Input data	Recall (%)	Precision (%)	F-measure (%)
1k	0.1	Platanus-allee	PE + 4 MP(≤15 kb)	88.852	98.974	93.640
		FALCON-Unzip	PacBio(x192)	**98.207**	97.607	**97.906**
		FALCON-Unzip, Pilon, PH	PacBio(x192) + PE	91.350	97.553	94.350
		Supernova	10X	95.130	**99.238**	97.141
	0.5	Platanus-allee	PE + 4 MP(≤15 kb)	**96.934**	**98.678**	**97.798**
		FALCON-Unzip	PacBio(x192)	84.578	97.192	90.447
		FALCON-Unzip, Pilon, PH	PacBio(x192) + PE	94.747	97.306	96.009
		Supernova	10X	93.869	98.186	95.979
	1.0	Platanus-allee	PE + 4 MP(≤15 kb)	**97.782**	**98.904**	**98.340**
		FALCON-Unzip	PacBio(x192)	97.731	96.919	97.323
		FALCON-Unzip, Pilon, PH	PacBio(x192) + PE	94.638	96.948	95.779
		Supernova	10X	92.032	97.650	94.758
	2.0	Platanus-allee	PE + 4 MP(≤15 kb)	**98.386**	**99.256**	**98.819**
		FALCON-Unzip	PacBio(x192)	97.728	97.073	97.399
		FALCON-Unzip, Pilon, PH	PacBio(x192) + PE	94.666	97.193	95.913
		Supernova	10X	68.728	98.230	80.873
5k	0.1	Platanus-allee	PE + 4 MP(≤15 kb)	74.022	93.223	82.520
		FALCON-Unzip	PacBio(x192)	**92.372**	91.893	92.132
		FALCON-Unzip, Pilon, PH	PacBio(x192)	82.751	91.768	87.027
		Supernova	10X	88.200	**97.314**	**92.533**
	0.5	Platanus-allee	PE + 4 MP(≤15 kb)	**91.213**	95.497	**93.306**
		FALCON-Unzip	PacBio(x192)	79.924	91.681	85.400
		FALCON-Unzip, Pilon, PH	PacBio(x192) + PE	89.417	91.769	90.578
		Supernova	10X	86.298	**95.634**	90.726
	1.0	Platanus-allee	PE + 4 MP(≤15 kb)	**92.963**	**95.801**	**94.361**
		FALCON-Unzip	PacBio(x192)	92.322	91.355	91.836
		FALCON-Unzip, Pilon, PH	PacBio(x192) + PE	89.372	91.351	90.351
		Supernova	10X	81.937	93.366	87.279
	2.0	Platanus-allee	PE + 4 MP(≤15 kb)	**94.616**	**96.822**	**95.706**
		FALCON-Unzip	PacBio(x192)	92.339	91.561	91.948
		FALCON-Unzip, Pilon, PH	PacBio(x192) + PE	89.421	91.694	90.543
		Supernova	10X	56.595	94.229	70.717

Supplementary Figures

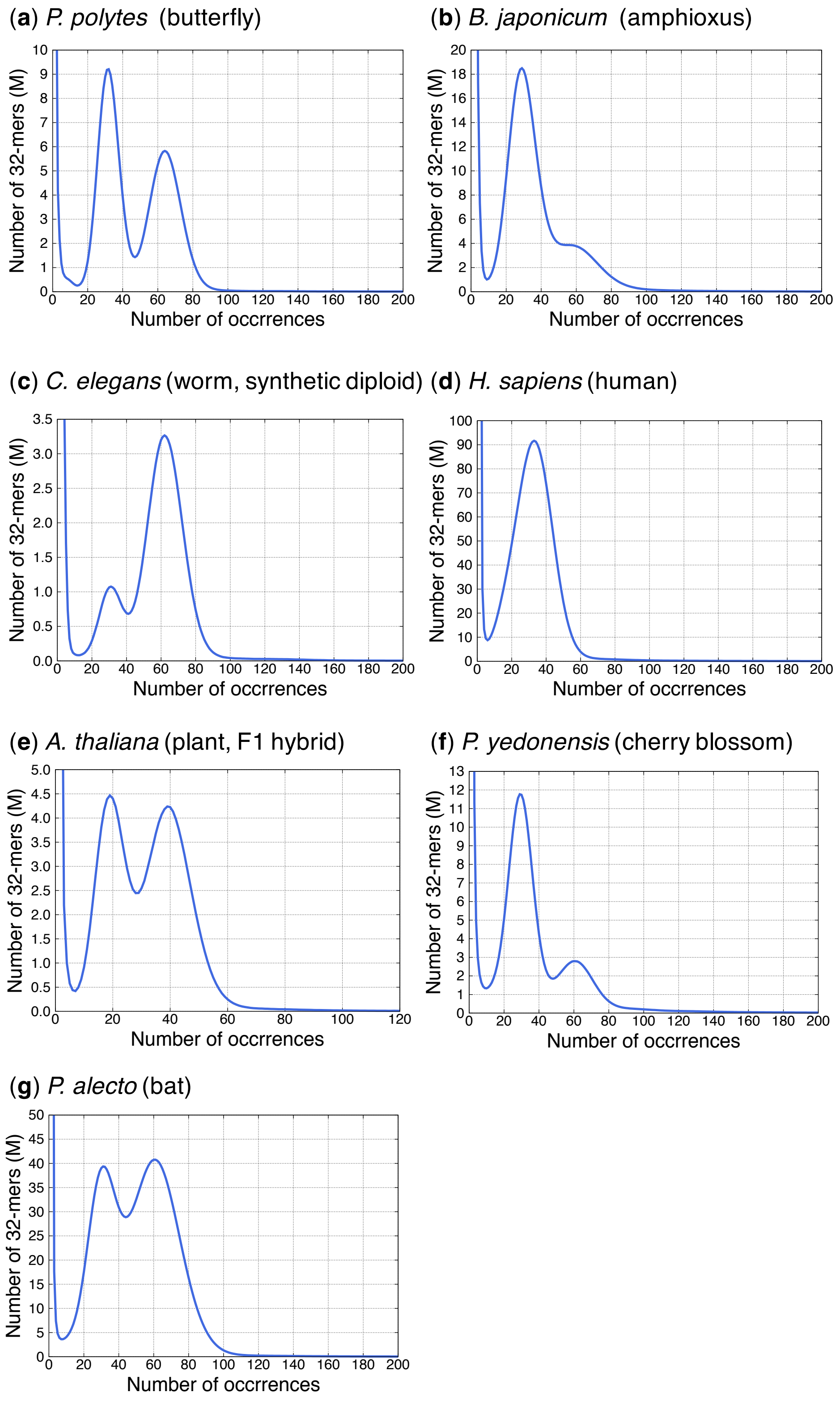

Supplementary Fig. 1. Distributions of the numbers of 32-mer occurrences.

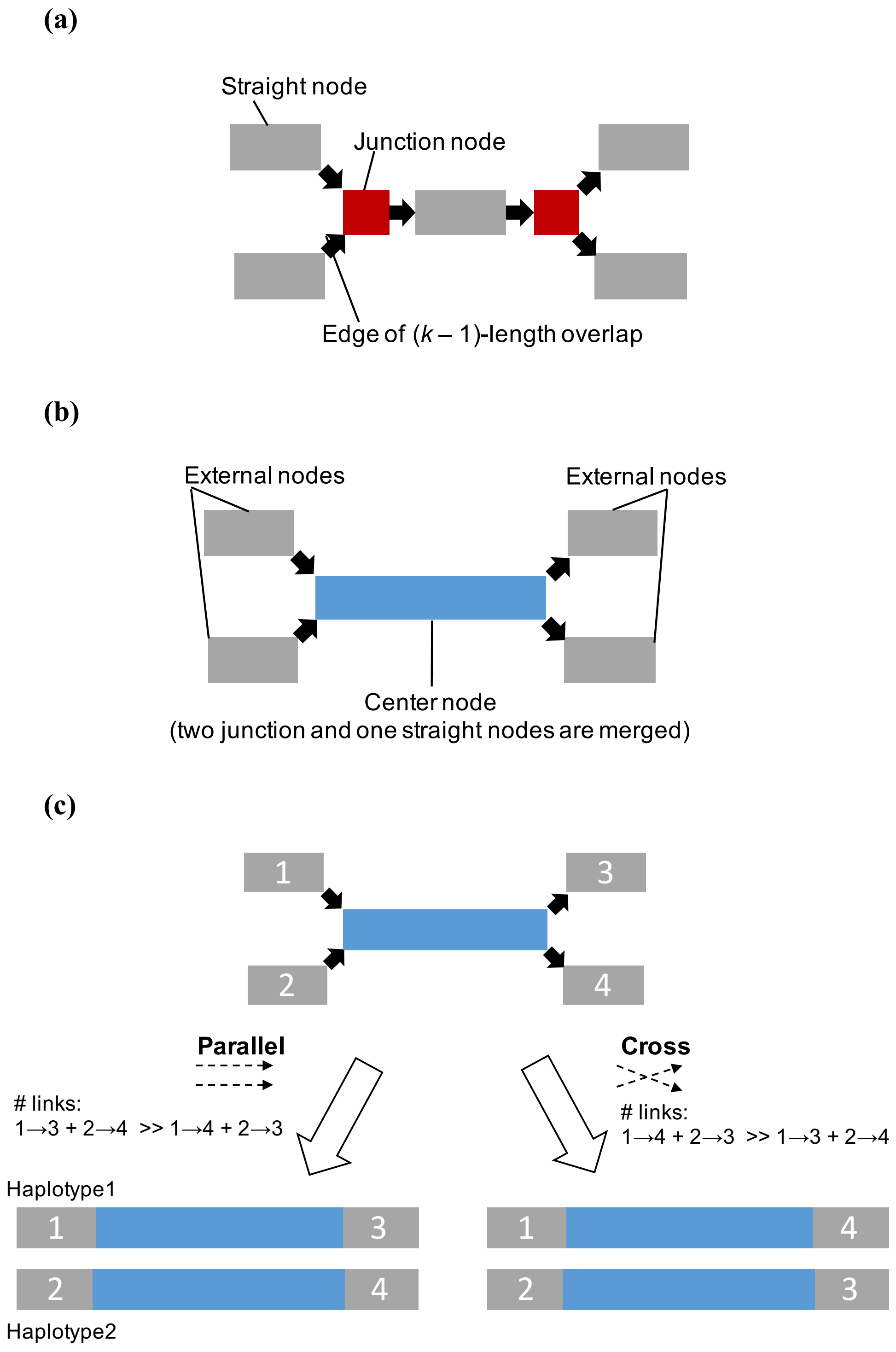

Supplementary Fig. 2. Schematic model of cross structures and untangling. (**a**) Raw cross structure in a de Bruijn graph. (**b**) Simplified cross structure in a de Bruijn graph. (**c**) Two types of untangling solutions.

(a)

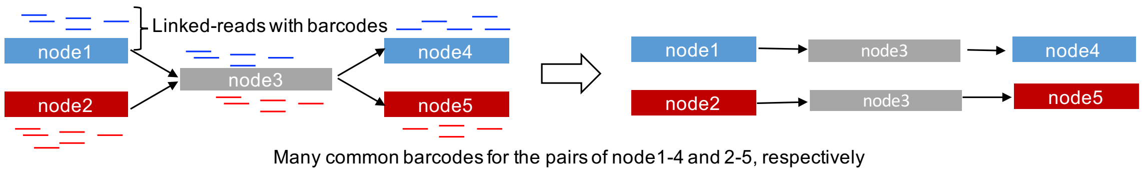

(b)

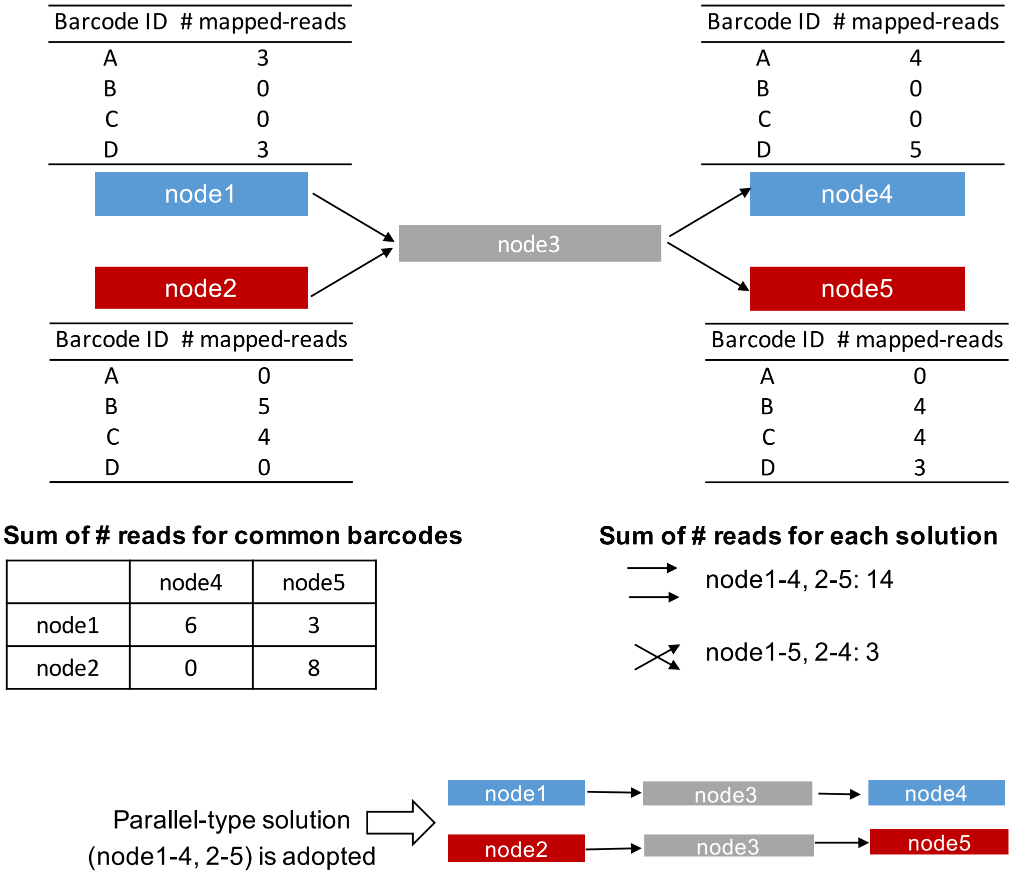

Supplementary Fig. 3. Untangling cross structure using linked-reads.

(a) Schematic model and (b) an example.

(a)

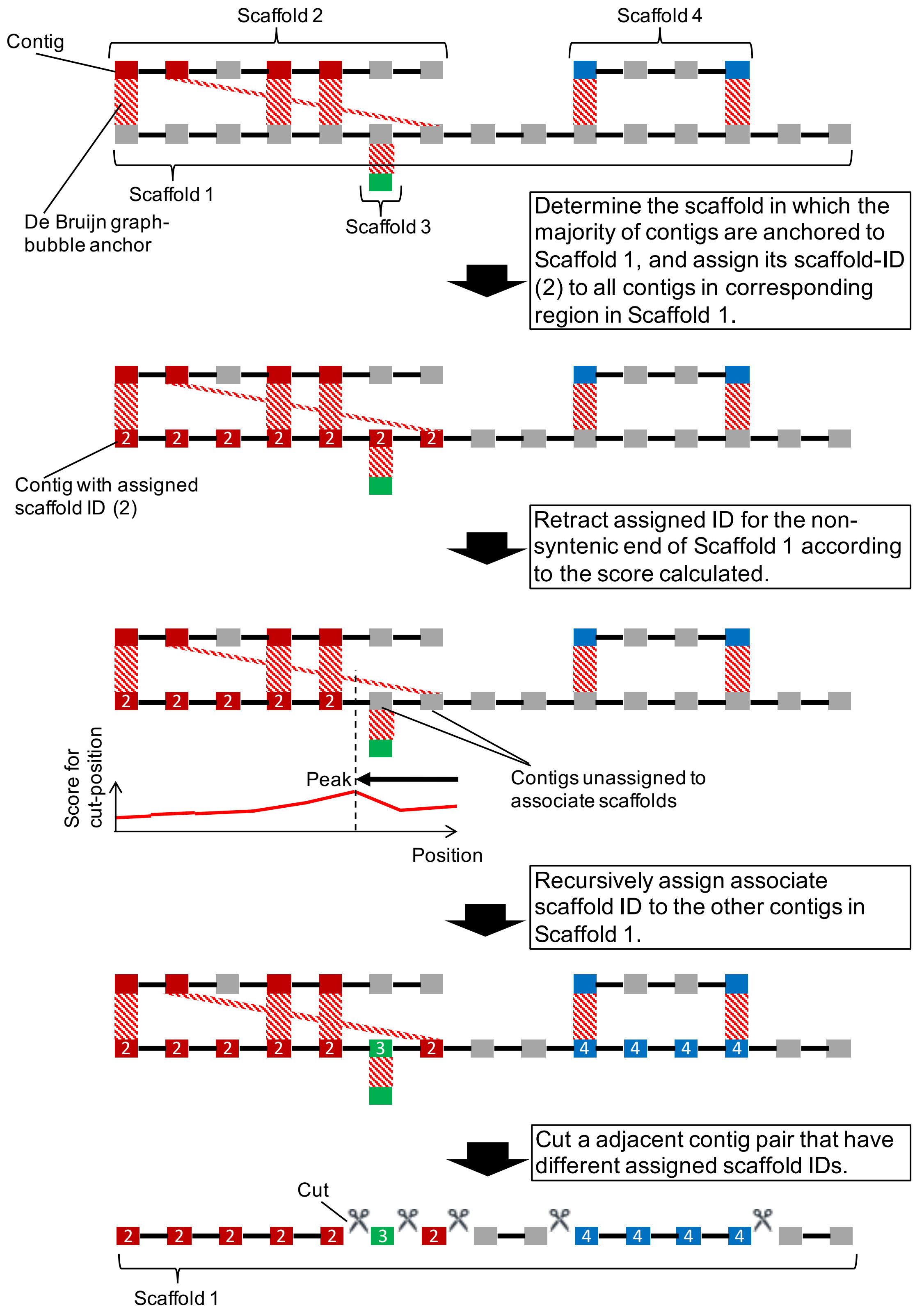

Scaffold 2
Scaffold 4
Contig
Scaffold 1
De Bruijn graph-bubble anchor
Scaffold 3
Determine the scaffold in which the majority of contigs are anchored to Scaffold 1, and assign its scaffold-ID (2) to all contigs in corresponding region in Scaffold 1.
Contig with assigned scaffold ID (2)
Retract assigned ID for the non-syntenic end of Scaffold 1 according to the score calculated.
Score for cut-position
Peak
Contigs unassigned to associate scaffolds
Position
Recursively assign associate scaffold ID to the other contigs in Scaffold 1.
Cut a adjacent contig pair that have different assigned scaffold IDs.
Cut
Scaffold 1

(b)

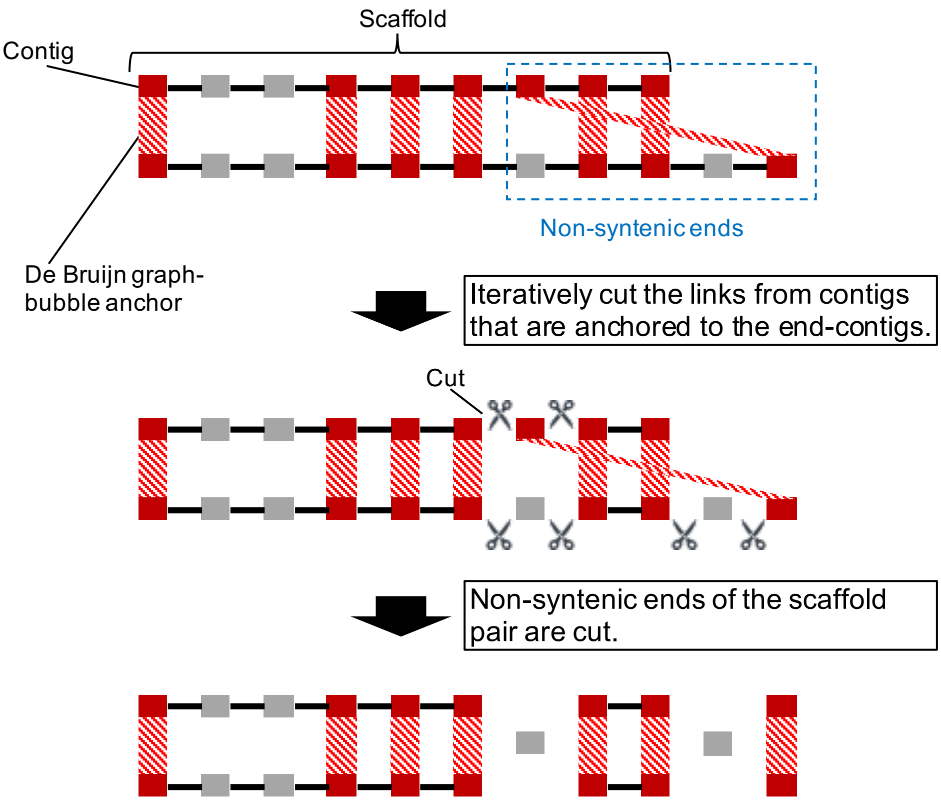

Supplementary Fig. 4. Haplotype synteny-based correction.

(a) Divisions at positions of error-candidates

(b) Processing of non-syntenic ends

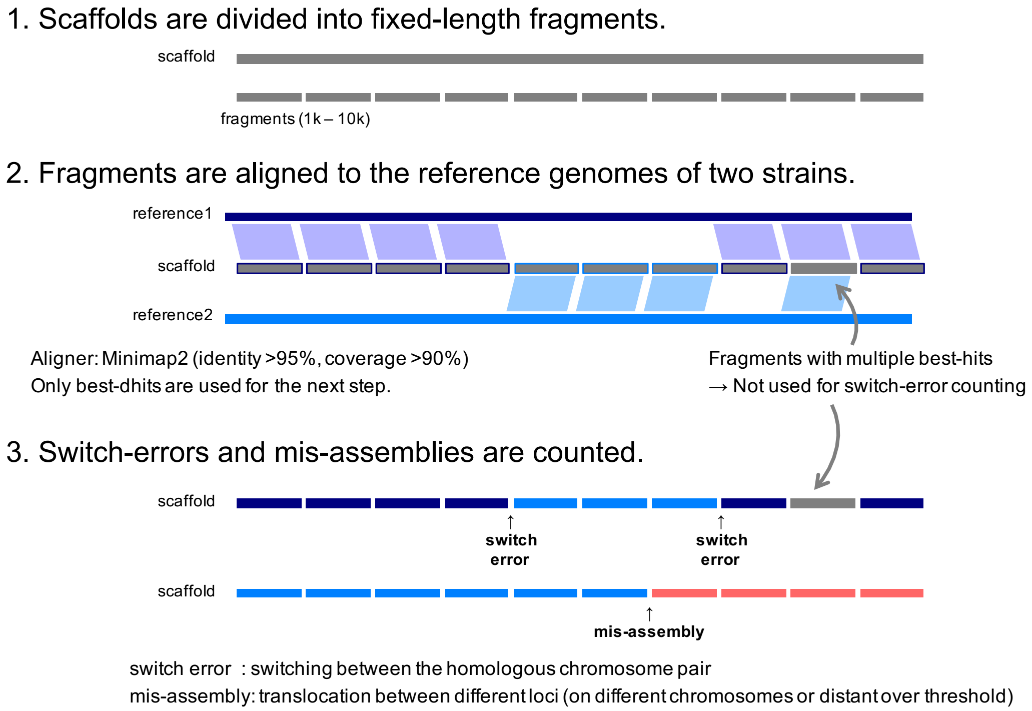

Supplementary Fig. 5. Schematic model of error detection for benchmarks.

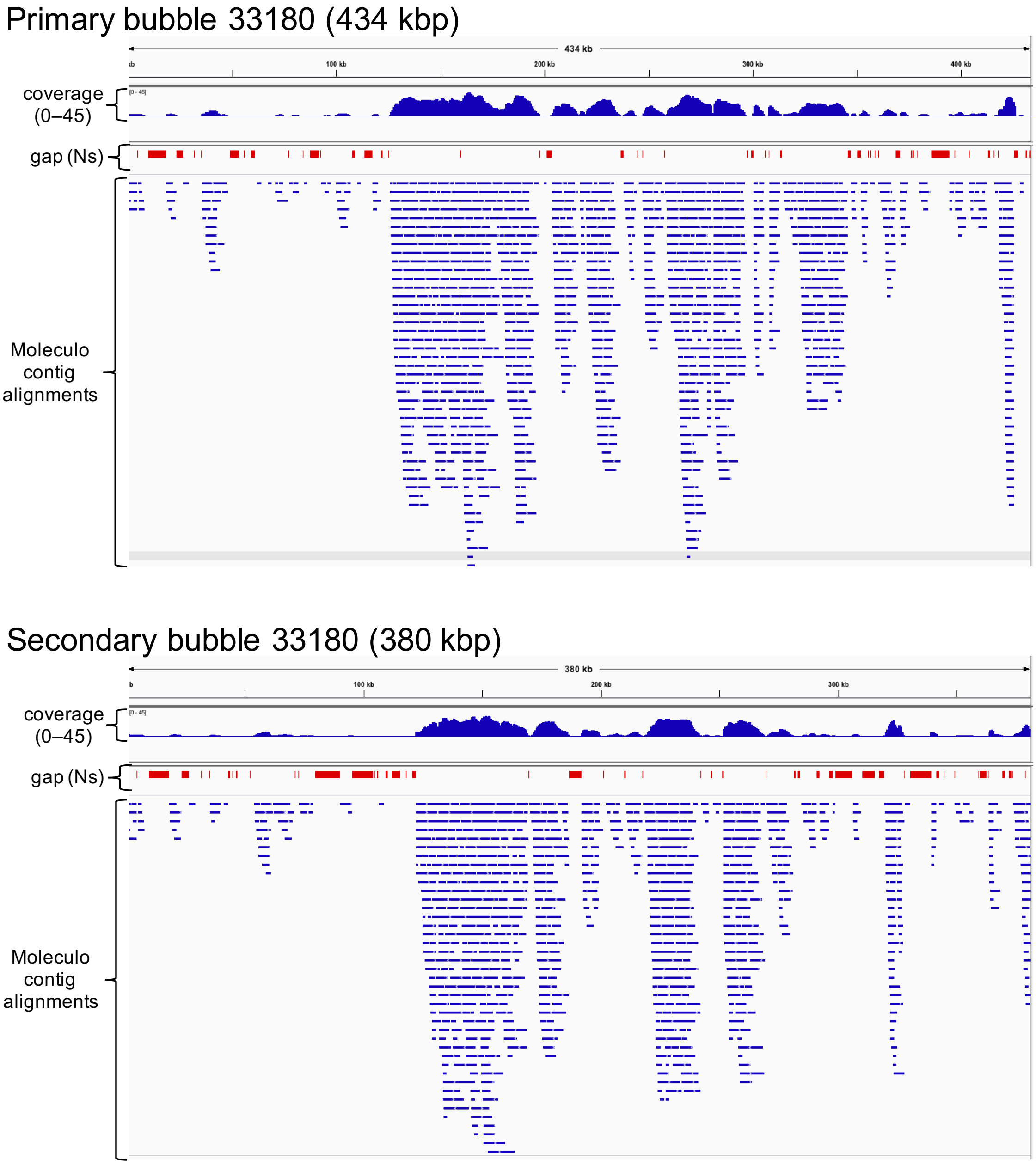

Supplementary Fig. 6. Moleculo contig alignment to the longest bubble absent in the reference (human).

The Moleculo contigs were aligned to all phased blocks of Platanus-allee by Minimap2, and the best hits (identity ≥ 0.99 and alignment-length > 1k) are shown.

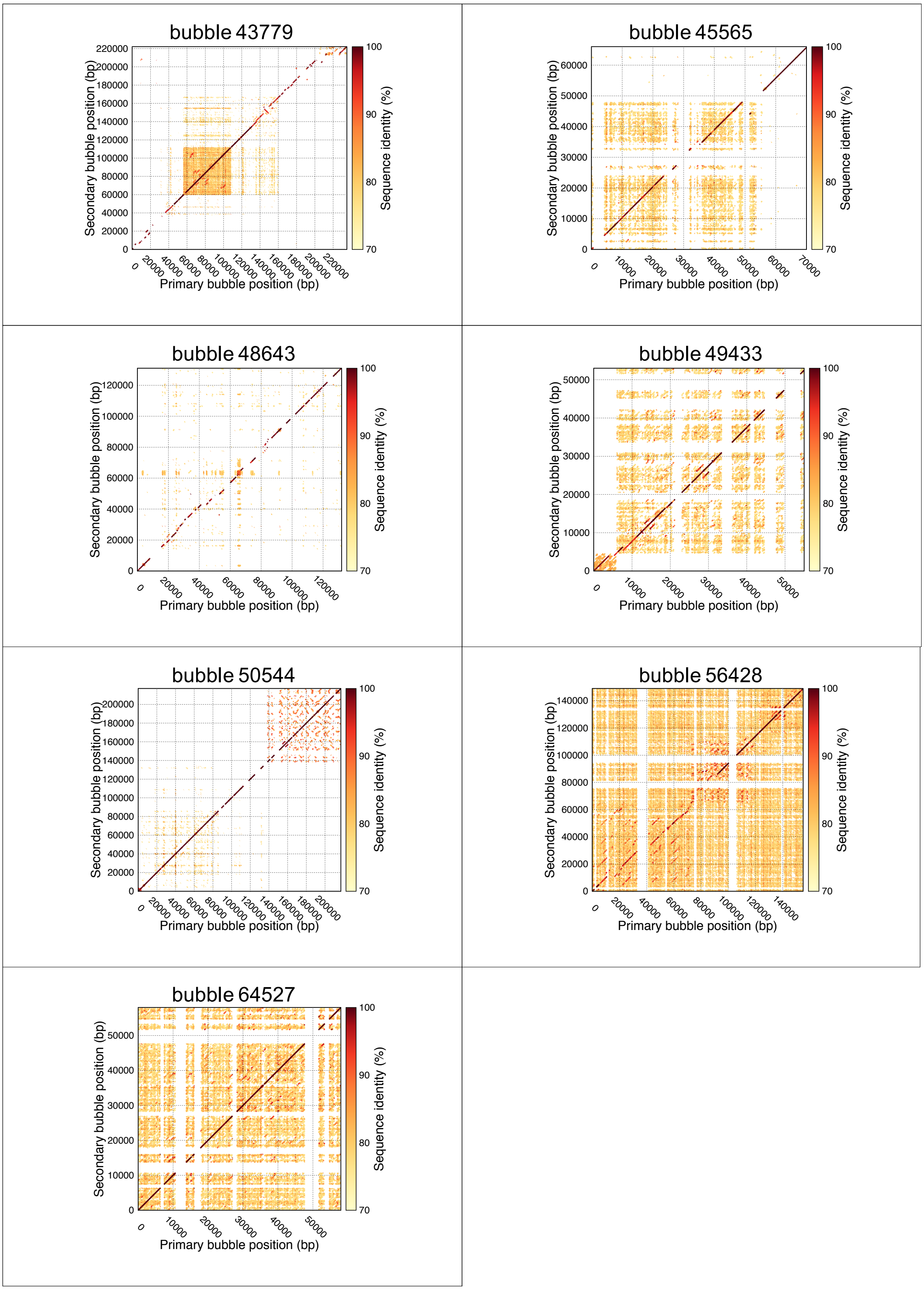

bubble 43779
bubble 45565
bubble 48643
bubble 49433
bubble 50544
bubble 56428
bubble 64527
Secondary bubble position (bp)
Primary bubble position (bp)
Sequence identity (%)

Supplementary Fig. 7. Examples of highly divergent and repeat-rich bubbles of Platanus-allee (human).

Alignments between the primary-bubbles and secondary-bubbles are shown. These bubbles were detected as those absent from the reference (see Methods, Detecting sequences absent from the human reference genome.

Supplementary References

1. Kajitani, R. *et al.* Efficient *de novo* assembly of highly heterozygous genomes from whole-genome shotgun short reads. *Genome Res.* **24,** 1384–95 (2014).
2. Chin, C.-S. *et al.* Phased diploid genome assembly with single-molecule real-time sequencing. *Nat. Methods* **13,** 1050–1054 (2016).
3. Weisenfeld, N. I., Kumar, V., Shah, P., Church, D. M. & Jaffe, D. B. Direct determination of diploid genome sequences. *Genome Res.* **27**, 757–767 (2017).
4. Koren, S. et al. *De novo* assembly of haplotype-resolved genomes with trio binning. **36**, 1174–1182 (2018).
5. Li, H. Minimap2: pairwise alignment for nucleotide sequences. *Bioinformatics* (2018). doi:10.1093/bioinformatics/bty191
6. Walker, B. J. *et al.* Pilon: An Integrated Tool for Comprehensive Microbial Variant Detection and Genome Assembly Improvement. *PLoS One* **9**, e112963 (2014).
7. Li, H. & Durbin, R. Fast and accurate short read alignment with Burrows-Wheeler transform. *Bioinformatics* **25,** 1754–1760 (2009).
8. Li, H. *et al.* The Sequence Alignment/Map format and SAMtools. *Bioinformatics* **25,** 2078–9 (2009).
9. Roach, M. J., Schmidt, S. A. & Borneman, A. R. Purge Haplotigs: allelic contig reassignment for third-gen diploid genome assemblies. *BMC Bioinformatics* **19**, 460 (2018).
10. Marçais, G. & Kingsford, C. A fast, lock-free approach for efficient parallel counting of occurrences of k-mers. *Bioinformatics* **27**, 764–770 (2011).
11. Vurture, G. W. *et al.* GenomeScope: fast reference-free genome profiling from short reads. *Bioinformatics* **33**, 2202–2204 (2017).
12. The *C. elegans* Sequencing Consortium. Genome Sequence of the Nematode *C. elegans*: A Platform for Investigating Biology. *Science* **282**, 2012–2018 (1998).
13. Thompson, O. A. *et al.* Remarkably Divergent Regions Punctuate the Genome Assembly of the *Caenorhabditis elegans* Hawaiian Strain CB4856. *Genetics* **200**, 975–89 (2015).
14. Kurtz, S. *et al.* Versatile and open software for comparing large genomes. *Genome Biology* **5**, R12 (2004).
15. Mostovoy, Y. *et al.* A hybrid approach for *de novo* human genome sequence assembly and phasing. *Nat. Methods* **13**, 587–590 (2016).

16. Eberle, MA. *et al.* A reference dataset of 5.4 million phased human variants validated by genetic inheritance from sequencing a three-generation 17-member pedigree. *Genome Res.* **27**, 157–164 (2017).
17. Dilthey, A. T. *et al.* High-Accuracy HLA Type Inference from Whole-Genome Sequencing Data Using Population Reference Graphs. *PLoS Comput. Biol.* **12**, e1005151 (2016).
18. Baek, S. *et al*. Draft genome sequence of wild *Prunus yedoensis* reveals massive inter-specific hybridization between sympatric flowering cherries. *Genome Biol.* **19**, 1–17 (2018).
19. Zhang, G. *et al*. Comparative analysis of bat genomes provides insight into the evolution of flight and immunity. *Science* **339**, 456–460 (2013).
20. Huang, W., Li, L., Myers, J. R. & Marth, G. T. ART: a next-generation sequencing read simulator. *Bioinformatics* **28**, 593–594 (2012).
21. Lau, B. *et al.* LongISLND: in silico sequencing of lengthy and noisy datatypes. *Bioinformatics* **32**, 3829–3832 (2016).
22. Luo, R., Sedlazeck, F. J., Darby, C. A., Kelly, S. M. & Schatz, M. C. LRSim: A Linked-Reads Simulator Generating Insights for Better Genome Partitioning. *Comput. Struct. Biotechnol. J.* **15**, 478–484 (2017).
23. Hu, X. *et al.* pIRS: Profile-based Illumina pair-end reads simulator. *Bioinformatics* **28**, 1533–1535 (2012).
24. Weisenfeld, N. I. *et al.* Comprehensive variation discovery in single human genomes. *Nat. Genet.* **46,** 1350–1355 (2014).
25. Sudmant, P. H. *et al.* An integrated map of structural variation in 2,504 human genomes. *Nature* **526**, 75–81 (2015).
